# Supplementary material for: Mental health and mental well-being of Black students at UK universities: a review and thematic synthesis
Source: BMJ Open. 2022 Feb 28;12(2):e050720. doi: 10.1136/bmjopen-2021-050720 (PMC8886426; doi:10.1136/bmjopen-2021-050720)
Supplement: Author's manuscript [file bmjopen-2021-050720.draft_revisions.pdf]

BMJ Open is committed to open peer review. As part of this commitment we make the peer review history of every article we publish publicly available.

When an article is published we post the peer reviewers' comments and the authors' responses online. We also post the versions of the paper that were used during peer review. These are the versions that the peer review comments apply to.

The versions of the paper that follow are the versions that were submitted during the peer review process. They are not the versions of record or the final published versions. They should not be cited or distributed as the published version of this manuscript.

BMJ Open is an open access journal and the full, final, typeset and author-corrected version of record of the manuscript is available on our site with no access controls, subscription charges or pay-per-view fees (http://bmjopen.bmj.com).

If you have any questions on BMJ Open's open peer review process please email info.bmjopen@bmj.com

BMJ Open

Mental Health and Mental Wellbeing of Black Students at UK Universities: A Review and Thematic Synthesis

Journal:	*BMJ Open*
Manuscript ID	bmjopen-2021-050720
Article Type:	Original research
Date Submitted by the Author:	02-Mar-2021
Complete List of Authors:	Stoll, Nkasi; King's College London Institute of Psychiatry Psychology and Neuroscience, Department of Psychological Medicine<br>Yalipende, Yannick; University College London, Division of Psychology and Language Sciences<br>Byrom, Nicola; King's College London Institute of Psychiatry Psychology and Neuroscience, Department of Psychology<br>HATCH, STEPHANI; King's College London Institute of Psychiatry Psychology and Neuroscience, Department of Psychological Medicine; King's College London, ESRC Centre for Society and Mental Health<br>Lempp, Heidi; King's College London Faculty of Life Sciences and Medicine, Department of Inflammation Biology
Keywords:	MENTAL HEALTH, QUALITATIVE RESEARCH, PSYCHIATRY, PUBLIC HEALTH, SOCIAL MEDICINE, EPIDEMIOLOGY

SCHOLARONE™
Manuscripts

I, the Submitting Author has the right to grant and does grant on behalf of all authors of the Work (as defined in the below author licence), an exclusive licence and/or a non-exclusive licence for contributions from authors who are: i) UK Crown employees; ii) where BMJ has agreed a CC-BY licence shall apply, and/or iii) in accordance with the terms applicable for US Federal Government officers or employees acting as part of their official duties; on a worldwide, perpetual, irrevocable, royalty-free basis to BMJ Publishing Group Ltd ("BMJ") its licensees and where the relevant Journal is co-owned by BMJ to the co-owners of the Journal, to publish the Work in this journal and any other BMJ products and to exploit all rights, as set out in our licence.

The Submitting Author accepts and understands that any supply made under these terms is made by BMJ to the Submitting Author unless you are acting as an employee on behalf of your employer or a postgraduate student of an affiliated institution which is paying any applicable article publishing charge ("APC") for Open Access articles. Where the Submitting Author wishes to make the Work available on an Open Access basis (and intends to pay the relevant APC), the terms of reuse of such Open Access shall be governed by a Creative Commons licence – details of these licences and which Creative Commons licence will apply to this Work are set out in our licence referred to above.

Other than as permitted in any relevant BMJ Author's Self Archiving Policies, I confirm this Work has not been accepted for publication elsewhere, is not being considered for publication elsewhere and does not duplicate material already published. I confirm all authors consent to publication of this Work and authorise the granting of this licence.

For peer review only

Mental Health and Mental Wellbeing of Black Students at UK Universities: A Review and Thematic Synthesis

Ms Nkasi Stoll*; nkasi.1.stoll@kcl.ac.uk; King's College London, Institute of Psychiatry Psychology and Neuroscience, Department of Psychological medicine, London, UK

E.319, Institute of Psychiatry Psychology and Neuroscience, Division of Academic Psychiatry, Psychological medicine, Camberwell, London, UK, SE5 8AF

Mr Yannick Yalipende; yannick.yalipende.20@ucl.ac.uk; University College London, Division of Psychology and Language Sciences, London, UK

Dr Nicola Bryom; Nicola.byrom@kcl.ac.uk; King's College London, Institute of Psychiatry Psychology and Neuroscience, Department of Psychology, London, UK

Professor Stephani Hatch*; stephani.hatch@kcl.ac.uk; King's College London, Institute of Psychiatry Psychology and Neuroscience, Department of Psychological medicine, London, UK; King's College London, ESRC Centre for Society and Mental Health, London, UK

Dr Heidi Lempp*; heidi.lempp@kcl.ac.uk; King's College London, Faculty of Life Sciences and Medicine, Department of Inflammation Biology, London, UK

Five keyword(s): MENTAL HEALTH**;** QUALITATIVE RESEARCH**;** PSYCHIATRY**;** PUBLIC HEALTH**;** SOCIAL MEDICINE**;** EPIDEMIOLOGY

Word count: 3824

For peer review only - http://bmjopen.bmj.com/site/about/guidelines.xhtml

Abstract

Background: There is a knowledge gap about the experiences that affect the mental health of Black university students in the UK. Current research is focused on understanding the continuation, attainment and progression gap between Black students and non-Black students. It is essential to know more about the interactions between personal and institutional factors on the mental health of Black students to explain the inequalities in their experiences and outcomes across the university lifecycle.

Objective: To thematically synthesise articles that explore the experiences that affect the mental health and mental wellbeing of Black university students in the UK.

Design: Qualitative thematic synthesis of a literature review.

Methods: We developed search strategies for four online databases (PubMed, Social Science Premium Collection via ProQuest, Open Access Theses and Dissertations, and Open Grey) covering January 2010 to July 2020. This search was combined with a manual search of reference lists and related citations. All articles in English addressing mental health and mental wellbeing experiences among Black university students studying at a UK university were included. A thematic synthesis was conducted to develop descriptive themes and analytical constructs.

Results: Twelve articles were included. Several themes were identified as affecting the mental health of Black university students in the UK: academic pressure, learning environment, Black gendered experience, isolation and alienation, culture shock, racism and support.

Conclusion: This review provides an appraisal of the factors affecting the mental health and mental wellbeing of Black students at UK universities, which need to be addressed by Higher Education policymakers and key decision-makers. Further research is needed about the mental health experiences of Black university students in relation to Black identities, suicidality, mental health language, the physical environment, and racism and other institutional factors.

For peer review only - http://bmjopen.bmj.com/site/about/guidelines.xhtml

Strengths and Limitations of this study

- To our knowledge, this is the first review and thematic synthesis to provide an overview of the personal and institutional experiences that affect the mental health and mental wellbeing of Black students studying at UK universities which can inform practice and policy.
- Recommendations for potential areas of priority in student mental health services design, development and reform provided.
- Although database and manual searching was extensive, it is possible that not all relevant studies were found due to inconsistent terminology for this topic and the racial category "Black" and "Mixed".

For peer review only

Introduction

There is increasing concern about the mental health and mental wellbeing of university students in the United Kingdom (UK)[1-4]. The university student lifecycle has been conceptualised in terms of: (i) the application experience which covers the interactions between potential students and the institution up to the point of arrival; (ii) the academic experience which encompass students' interactions with their institution of study; (iii) the campus experience, which includes students' life not directly connected with study and may include activities away from the campus; and (iv) the graduate experience which covers the institution's role in assisting students' transition to employment or further study[5].

Throughout the lifecycle, students are exposed to a range of experiences that make university a high-risk period for maladaptive coping and possible onset of poor mental health. These experiences include (but not limited to): individuation, separation from family, new social connections, increased autonomy and responsibility, academic-related stress, financial concerns, sleep disruption, balancing conflicting demands of studying with personal and family life, and exposure to risky behaviours, including recreational drug use and alcohol binging[6-10].

Late adolescence through young adulthood is a peak age period for the first onset of mental health problems[11], which overlaps the period that many young adults enter and navigate the university student lifecycle. Unsupported mental health problems are associated with progression to other co-morbid disorders, substance use disorders, self-harm and suicide ideation and attempts[12-15]. According to Higher Education Statistics Agency, there are 2.4 million university students in higher education in the UK[16]; which means that universities are in the best position to provide prevention and intervention to many young adults during a critical transitional period.

In the UK, despite Black students being more likely than White students to engage and participate in their university studies[17], they report lower satisfaction and are less likely to complete their course, achieve a good degree (1st or 2:1), and progress to further education[18]. In addition to the aforementioned experiences and risk factors for poor mental health, there is tentative research

For peer review only - http://bmjopen.bmj.com/site/about/guidelines.xhtml

around the institutional issues affecting Black university student mental health including: inequality in access, under-representation, the attainment gap, institutional racism, and de-colonising the curriculum[19-23]. To explain the inequalities in student experiences and outcomes across the university lifecycle, further understanding of the interactions between personal and institutional factors on the mental health of Black students' is needed.

This review aims to synthesise existing studies that explore mental health and mental wellbeing in Black students at UK universities. Therefore, our research question is as follows: what are the experiences that affect the mental health and mental wellbeing of Black university students in the UK, as reported in the literature?

Methods

Design

A review of the literature including quantitative, qualitative and mixed studies was conducted on peer reviewed and non-peer reviewed articles, addressing mental health and mental wellbeing of Black students at UK universities. Reporting of this review was guided by the Enhancing Transparency of Reporting the Synthesis of Qualitative Research framework, as the included articles were all qualitative[24].

Search methods

The following databases were searched for the period of January 2010 to July 2020: PubMed, Social Science Premium Collection via ProQuest, Open Access Theses and Dissertations, and Open Grey. The search strategy included several combinations of the following medical subject heading (MeSH) terms and keywords[25]: "mental health" or "psychological distress" or "mental wellbeing" and "University student" and "Black Caribbean" or "Black African" or "Black Mixed" or "Black ethnic*".

For peer review only

Data collection

All search results were saved to Zotero bibliographic management software and duplicates were removed. To be included in the review, articles had to (1) focus on the mental health or mental wellbeing of Black students at UK universities (2) apply empirical (e.g., quantitative, qualitative, or mixed-method research) or non-empirical research methods (e.g., theoretical, opinion-based, student transcripts, policy papers) (3) be written in English, (4) be conducted in the UK. The first author (NS) removed all articles that did not fit the inclusion criteria after full-text review. NS manually-searched reference lists of eligible articles and contacted lead authors to search for unpublished literature. The authors took the decision to be lenient in the inclusion criteria during the screening and inclusion of articles because existing research on Black university student mental health is limited, and to enable the inclusion of articles that add value to the discussion of the topic. All included articles were critically appraised by NS and the author YY using the 'Critical Appraisal Skills Programme' (2018)[26] checklist for qualitative research.

Data analysis and synthesis

For each article, all text from 'Results/Findings' and 'Discussion' were extracted and imported into NVivo V.12 software (NVivo Qualitative data analysis Software; QSR International, V.12, 2018). Study characteristics were extracted into a Microsoft Excel grid by NS and revised by YY. Full text screening, selection, data extraction and critical appraisal of all articles was conducted prior to data analysis. A thematic analysis was conducted to describe and compare the main findings, following Braun and Clarke's (2006)[27] six-step guide. NS carefully read each article to familiarise themselves with the content, annotating initial ideas for codes and themes. NS then read and re-read the articles to develop an initial coding scheme, using a constant comparative approach[28]. Themes and sub-themes were identified to capture important patterns across and within the articles included. Six articles (50% of the sample) were randomly selected to be coded independently by YY, to provide validation. The codes were examined for similarities and differences, and then organised into a hierarchy to create the final codes. Any differences were resolved by SH. The final codes were validated by NS, YY, SH, and HL, before being synthesized

For peer review only - http://bmjopen.bmj.com/site/about/guidelines.xhtml

into the final thematic framework presented in this paper to minimise researcher subjectivity and improve the credibility of the work.

Patient and Public Involvement

The lead author is a Black Caribbean PhD student, and the second author is a Black African master's student. Both were involved in the design, conduct, reporting, and dissemination of this research.

Results

Included studies

Figure 1 shows the number of articles identified and rejected at each stage of the review process[29]. The search strategies identified 388 references of which 369 were kept after removing duplicates. After screening, 87 articles were read completely. Of these, 75 articles were excluded. A final sample of n=12 studies were included. Table 1 presents the characteristics of the included studies.

Table 1 Characteristics of included studies (in date order)

**Author(s)**	**Database**	**Type of article**	**No. of Black participants**	**Subject Characteristics**	**Location**	**Psychological phenomena explored**	**Methods**
Myrie, C. V. and Gannon, K. (2013)	Hand searched	Journal article	3	2 Black African Students, 1 Black African and Caribbean Student	East and North London	Wellbeing	Interviews
Jones, C. (2017)	PubMed	Blog	1	Black academic staff	UK	Wellbeing	Personal reflections and notes
Alloh, F. T., Tait, D., & Taylor, C. (2018)	Proquest	Journal article	9	International Nigerian students studying at the master's-level in a U.K. university	southeast of England	Wellbeing	Interviews

For peer review only - http://bmjopen.bmj.com/site/about/guidelines.xhtml

Arday, J. (2018)	Proquest	Journal article	14	32 BME university students	UK	Mental Health	Interviews focus Groups
Baxe, K. (2018)	PubMed	Conference notes	1	Black students	UK	Mental Health	Personal Reflections And Notes
Crittle, K. (2018)	Proquest	Blog	1	African American student	UK	Stress	Personal Reflections And Notes
Cummings, S. (2018)	Open Grey	Doctoral thesis	5	Black women with Caribbean heritage, who were being educated at a university in the East Midlands	East Midlands	Wellbeing	Focus Groups
Akel, S. (2019)	PubMed	Report	Not specified	195 BME students. 'Black' refers to people of African heritage and the diaspora.	London	Wellbeing	Interviews focus Groups
Bhatti, S. (2019)	Open Grey	Doctoral thesis	5	Black students	London	Body image	Focus Groups
Bunce, L., King, N., Saran, S., & Talib, N. (2019)	PubMed	Journal article	14	14 full-time students from two degree programmes in health and social care related subjects - Black African (12) and White and Black Caribbean (2)	Unknown	Wellbeing	Focus Groups
Hayford, A. (2019)	Hand searched	Dissertation	6	One was Caribbean, one was Somalian, one was Nigerian, two were mixed black African, and one was Ghanaian	East Anglia	Wellbeing	Interviews
Jackson-Cole, D. (2019)	Open Grey	Doctoral thesis	10	Postgraduate students. two Black or Black British – Caribbean participants, seven Black or Black British –African, one Mixed –White and Black African	England	Wellbeing	Interviews

For peer review only - http://bmjopen.bmj.com/site/about/guidelines.xhtml

All included articles were assessed by NS and YY as reasonable quality and were therefore judged to have relevant contributions for the thematic synthesis (Table 2).

Table 2 Critical Appraisal Skills Programme (CASP) Checklist for included articles (in date order)

	Was there a clear statement of the aims of the research?	Is a qualitative methodology appropriate?	Was the research design appropriate to address the aims of the research?	Was the recruitment strategy appropriate to the aims of the research?	Was the data collected in a way that addressed the research issue?	Has the relationship between researcher and participants been adequately considered?	Have ethical issues been taken into consideration?	Was the data analysis sufficiently rigorous?	Is there a clear statement of findings?	How valuable is the research?
Myrie, C. V. and Gannon, K. (2013)	Yes	Yes	Yes	Yes	Yes	No	Yes	Yes	Yes	Valuable
Jones, C. (2017)	No	N/A	N/A	N/A	N/A	N/A	N/A	N/A	Yes	Valuable
Alloh, F. T., Tait, D., & Taylor, C. (2018)	Yes	Yes	Yes	Yes	Yes	No	Yes	Yes	Yes	Valuable
Arday, J. (2018)	Yes	Yes	Yes	Yes	Yes	No	Can't Tell	Yes	Yes	Valuable
Baxe, K. (2018)	No	N/A	N/A	N/A	N/A	N/A	N/A	N/A	Yes	Valuable
Crittle, K. (2018)	Yes	Yes	Yes	Yes	Yes	Yes	Yes	Yes	Yes	Valuable
Cummings, S. (2018)	Yes	Yes	Yes	Yes	Yes	Yes	Yes	Yes	Yes	Valuable
Akel, S. (2019)	Yes	Yes	Yes	Yes	Yes	No	Yes	Yes	Yes	Valuable
Bhatti, S. (2019)	Yes	Yes	Yes	Yes	Yes	Yes	Yes	Yes	Yes	Valuable
Bunce, L., King,	Yes	Yes	Yes	Yes	Yes	No	Can't Tell	Yes	Yes	Valuable

For peer review only - http://bmjopen.bmj.com/site/about/guidelines.xhtml

N., Saran, S., & Talib, N. (2019)									
Hayford, A. (2019)	Yes	Yes	Yes	Yes	Yes	Yes	Yes	Yes	Yes	Valuable
Jackson-Cole, D. (2019)	Yes	Yes	Yes	Yes	Yes	Yes	Yes	Yes	Yes	Valuable

Synthesised findings

The concept matrix for the findings can be seen in Table 3.

Table 3 Concept matrix for included studies (in date order)

	**Support**	**Isolation and Alienation**	**Racism**	**Culture Shock**	**Black gendered experience**	**Learning environment**	**Academic pressure**
Myrie, C. V. and Gannon, K. (2013)	X	X			X		
Jones, C. (2017)	X		X	X			X
Alloh, F. T., Tait, D., & Taylor, C. (2018)	X		X	X			X
Arday, J. (2018)	X	X	X		X		X
Baxe, K. (2018)		X					
Crittle, K. (2018)	X	X					
Cummings, S. (2018)	X	X	X		X	X	
Akel, S. (2019)		X	X			X	
Bhatti, S. (2019)	X				X		
Bunce, L., King, N.,		X		X		X	

For peer review only - http://bmjopen.bmj.com/site/about/guidelines.xhtml

For peer review only

Saran, S., & Talib, N. (2019)							
Hayford, A. (2019)	X	X		X			
Jackson-Cole, D. (2019)	X		X		X	X	

Academic pressure

In six out of 12 articles, academic pressure was a determinant of mental distress for Black students[30-35]. Students described their mental health being adversely affected by the multiple and simultaneous academic tasks they were expected to complete during their degree[30-31]. Cumulatively, Black students reported that unlike their non-Black peers, they faced pressures from their families and the wider Black community which meant they needed to work harder to succeed at university; which further exacerbated mental distress[30,31,33,34]. This pressure came from their families continuously reminding them of the sacrifices and investments made, specifically immigration and financial strain[30,31,35]. Black students with pre-existing mental and physical health conditions might be especially vulnerable to poor mental health caused by this academic pressure[31-32]. There was some evidence that Black students might not seek mental health support and instead prioritise their continuation and success in academia, at the expense of their mental health, as a mental illness diagnosis might affect their success in academia[30,33].

For peer review only

Learning environment

In four of the 12 articles, researchers described Black students' perceptions of the university learning and teaching environment and how it impacted their mental health and mental wellbeing[34, 36-38]. Lectures which had discussions on diversity, inclusion, ethnicity, race, and identity were said to be cathartic and liberating for Black students, which contributed to positive wellbeing[36-38]. Black students reported they had to censor themselves in academic spaces to be seen by White students and staff as acceptable and agreeable instead of loud, disruptive and confrontational[37]. This behaviour was believed to improve their learning experience and relationship with teaching staff, at the expense of their wellbeing. Participants spoke about their lack of relatedness to the White

For peer review only - http://bmjopen.bmj.com/site/about/guidelines.xhtml

majority academic staff, students, and teaching materials and racism in the classroom made them feel excluded, frustrated, distressed, discouraged and unmotivated to engage in their degree course[34,36-38]. In university seminars Black people were racially stereotyped as bad people, criminals, with lower intellectual ability by White students, and these stereotypes were sometimes reinforced by course materials[36-38]. Black female-identifying students recounted their academic knowledge was over-policed and over-scrutinised by teaching staff, inaccessibility of learning and wellbeing support compared to men and White women on their course, and the impact this had on their mental health and learning experience[34].

Black gendered experience

The Black student mental health experience of being at the intersection between race and the oppressive structures of traditional masculinities and femininities was discussed in five articles[33-35,37,39]. Black male-identifying students reported being affected by the discourses of hyper-masculinity, which put pressure on them to not show their emotions, cope with their mental distress and continue with their studies on their own[33,35]. This caused them to feel shame when seeking mental health support. For some female-identifying Black students, feelings about their health, ideal body shape and size were influenced by the beliefs, culture, race, and ethnicity of the people in their lives[39]. The importance of seeing their own body shape among Black students who looked like them was discussed. Some students felt the White body ideal is not the same as the Black body ideal, therefore visiting their country of origin (the Caribbean), positively impacted their stress levels, self-esteem and body image. Social media and the music industry were mentioned as vehicles in which the ideal Black female body is narrated, scrutinised and picked apart[39].

Isolation and Alienation

Eight out of 12 included studies discussed isolation and alienation[30,33,35-38,40-41]. Black students who struggled with their mental health experienced stigma from the Black community before attending university, leaving them isolated[30]. At university, participants reported being ignored or avoided by their non-Black peers in learning, social, and living environments which led to feelings of lack of belonging, diminished their overall wellbeing and evoked a range of negative emotions,

For peer review only
For peer review only - http://bmjopen.bmj.com/site/about/guidelines.xhtml

including discomfort, distress, frustration, and anger[30,33,36-38,40-41]. Those with little or no mental health support from family felt isolated and alienated from their friends, family, peers and academic staff, and used denial to cope with the lack of support, which exacerbated their mental health problems[33].

Black students reported that a lack of relatedness to White students affected their wellbeing, undermined their motivation for academic success and had a negative influence on their sense of autonomy and competence[30,36-38]. Black students mentioned White students did not understand that they have to re-construct their identity in the White image and practice silence to be successful at university and that this process negatively affected their mental health[30].

Participants in one article discussed that academic staff do not always create safe and protective learning and living spaces for Black university students[38]. As a result, Black students reported they had to defend themselves, which made White students uncomfortable, leaving Black students feeling bullied, alienated and alone. Participants who had the support of other Black students in their learning environment at university reported being more comfortable, understood, and happy[36]. However, this may not apply to Black students who are international students. One article reported a student being in tears, feeling alone and isolated, and almost dropping out of university due to their African accent isolating them from Black British students and non-Black students[36].

Culture Shock

There was evidence suggesting that Black international-status students had to adjust to new environments, cultures, and lifestyles in the UK which was characterised by changes in diet, weight, physical activity, alcohol and smoking consumption, and led to stress, depression, loneliness, and loss of confidence[30-32, 36]. Black students who emigrated from a collectivist culture to the UK experienced culture shock that was emotionally stressful[32]. Contrastingly, there was evidence[32] to suggest Black home-status students had slightly poorer mental health and lower self-esteem than Black international-status students. Indeed, one article reported some Black international-status students had higher stress levels in their home country which gave them the perspective of living in the UK as being less stressful[32].

For peer review only - http://bmjopen.bmj.com/site/about/guidelines.xhtml

Racism

Five of the 12 included studies explored how institutional racism, discrimination, and hegemonic White privilege made universities toxic spaces for Black students', which affected their mental health and wellbeing[31, 33-34, 37-38]. The constant stress of being confronted with racism (including racial microaggressions), discrimination, and having to survive hostile racist environments at university led to poor mental health and mental illness in Black students[31,33-34,37]. Racism had negative consequences on Black students' sense of belonging at university, motivation to socialise, their interactions with White students and staff, and their academic achievements and progression; which led to further mental distress. Black students' recognition of racial difference had a greater impact on their mental wellbeing and potential or experienced mental health difficulties, than evidence that others treated them differently on account of their race[37]. Participants felt unable to express their feelings of racism and difference from White peers to others for fear they would not be understood, would be judged or rejected by peers and university staff, or due to uncertainty that these feelings were valid or legitimate[37]. These unarticulated feelings resulted in disengagement from university study and services.

Support

In addition to the aforementioned factors possibly influencing the mental health of Black students, mental health support was also discussed[30-35, 37-40]. Black students reported they had to take care of their own mental health by trying to remain positive and use distraction, research psycho-educational resources, change daily habits including exercise and nutrition, and use food to cope with stress at university and connect them to family[32-34, 37, 39].

Access to mental health support from family, friends, and peers was identified as a contributory factor to positive wellbeing and success at university[30-31,33-34,37,40]. One article reported Black students who did not have familial social capital found accessing university mental health support more difficult[33]. Four included articles[31,33, 35,40] had findings linked to mental health stigma and taboo leaving Black students feeling unsupported by their family at university and unable to get

For peer review only - http://bmjopen.bmj.com/site/about/guidelines.xhtml

the professional support needed. Black-only spaces at university were reported as important to obtain support for mental and wellbeing[30-31, 33, 37, 40]. These spaces gave students comfort, where they were not judged and misunderstood bv non-Black students and staff and could be with students from a shared background and experience. These Black-only spaces were especially important when there was a lack of Black students in classroom spaces; and could be online or in person. Black students also placed importance on mental health support from an understanding religious organisation or chaplain at their university[30-31,33].

Included articles described Black students' beliefs that university services were institutionally racist, treated Black students as a monolithic group, thereby failed to provide appropriate services; and did not take responsibility for this[30-31,33,38]. Participants' awareness that White mental health practitioners at university services were the majority race prevented them from accessing services, because of perceived racial stereotyping and lack of trust[33,38]. Some Black students avoided university mental health services to such an extent that they were not knowledgeable about what was available for them[30]. Black students who needed mental health support did not seek such services until in crisis[30,33] and when they did, faced long wating times[33]. Black students who did receive help from university mental health services reported experiencing racialised stereotypes that affected their relationship with White mental health practitioners[30-31,33,38,]. They had to educate practitioners on Black culture and the Black experience (which included how racism and micro-aggressions affected their mental health), which was a burden on their mental health and wellbeing[30-31,33,38]. Because of this, students felt more comfortable with a Black, Asian or other minoritised ethnic mental health practitioner[30,33,38]. Black students described a sense of helplessness regarding their mental healthcare treatment[30,33,38]. One article discussed Black students felt unable to challenge mental health professionals' about their healthcare treatment because they may be labelled as difficult or unstable, and worsen their relationship with the mental healthcare provider[33].

Discussion

For peer review only - http://bmjopen.bmj.com/site/about/guidelines.xhtml

This review and thematic synthesis provides evidence on how the following experiences affect the mental health and mental wellbeing of Black university students in the UK: academic pressure, learning environment, Black gendered experience, isolation and alienation, culture shock, racism and support. The literature largely demonstrates an interest in Black university student mental health but is generally lacking in exploration on whether and how mental health experiences differ across different Black student identities (including gender, culture, ethnicity, nationality) and social statuses. This is important given the finding that Black students believe universities treat them as a monolithic group[30-31,33,38]. There is a knowledge gap on suicidality among Black students. A recent national study[50] found the risk of suicide is lower amongst Black university students compared to White students (risk ratio (RR) 0.53 (95%CI 0.32–0.88)). This finding needs to be investigated further. One article included in this review[30] discussed that mental health is either not spoken about or spoken about differently in the Black community compared to within the wider UK university community. More studies are needed on mental health language and literacy.

The review findings highlight that for Black university students', their mental health and mental wellbeing may be deeply rooted in institutional factors or dimensions, largely racism[31,33-34,37-39], which negatively affects most aspects of their higher education experiences, particularly in the learning, social, and living environments. Critical Race Theory in Education (CRT-E) is used to engage with and work against racism in the context of UK higher education[23,42-43]. CRT-E theorists have explored racial inequality in admissions, curriculum and pedagogy, teaching and learning, institutional culture, campus racial climate, and policy and finance[42-49]. However, CRT-E has failed to interrogate the role mental health plays in racial inequality. The Office for Students[2] stated that "black students with mental health conditions are being failed throughout the student cycle" (pg. 6), evidenced by their data which shows Black full-time students who report a mental health condition have some of the lowest attainment, continuation, and progression rates[2]. To understand and begin to address these concerns, further research into the mental health experiences of, risk factors for and challenges faced by Black university students is needed to understand how the historically racist systems embedded within UK higher education institutions create racial inequalities in student experiences, outcomes, and progression disadvantaging Black students.

For peer review only - http://bmjopen.bmj.com/site/about/guidelines.xhtml

This review is very timely given the recent global Black Lives Matter movement and protests following the death of George Floyd. Growing conversations around the role race and institutional racism play on the mental health of Black people motivated students and staff to write multiple open letters[51-56] demanding UK universities do more to tackle racism, be anti-racist, and diversify and decolonise their curricula and institutions. Students and staff are calling out for UK universities to provide racially and culturally appropriate mental health support for their Black students. However, to achieve this, further research is needed to interrogate the euro-centric, ethno-centric and ego-centric ways in which institutional policy, research and mental health practices disadvantage different Black student from varying identities and social statuses across the university student lifecycle.

The results of this review can assist academic staff, researchers, funders and policy makers to identify potential areas of priority in student mental health services design, development and reform with Black students. Based on the review findings, the authors recommend further investigation into the following potential strategies: (a) in-person or virtual Black student mental health peer support groups; (b) decolonization of mental health support services; (c) race, racism and mental health training for academic staff and mental health practitioners; (d) decolonization of the classroom environment and teaching and learning materials; (e) scholarships and support funds for Black students[57]; (f) involvement of family in mental health support; (g) improve knowledge and accessibility of available services; (h) train and employ Black mental health professionals; (i) involve religious organisations and chaplains in mental health support; (j) targeted mental health support for international-status students.

Strengths and limitations

To our knowledge this is the first review and thematic synthesis to summarise the available literature on the experiences that affect the mental health and mental wellbeing of Black students studying at UK universities. By summarising these results, we make this information more readily available to students, academic staff, policymakers and researchers. An exhaustive literature search was conducted using multiple databases, and manual searching. Dissertations, blogs and

For peer review only - http://bmjopen.bmj.com/site/about/guidelines.xhtml

unpublished articles were included alongside published articles. Our analysis allowed us to extract multiple recurrent themes pertaining to Black students' mental health. The results focus on both the personal and institutional factors that influence the mental health of Black university students.

The review has the following limitations. Despite the study selection criteria, no quantitative studies were included as they did not report the mental health experiences of Black university students. Secondly, although database and manual searching was extensive, it is possible that relevant articles were missed due to inconsistent terminology for mental health and mental wellbeing and the racial category "Black". Thirdly, the authors were unable to fully capture Black students' experiences as the citations selected from the included articles are a sample of the participants' subjective experiences and of the researchers' own analyses. Fourthly, articles which included Black, Asian and minoritised ethnic participants did not always clearly differentiate citations and themes derived specifically from Black student responses. The mixed racial category was not clearly defined in some articles so students who were mixed Black heritage may have been missed in the analysis. Responses had to be drawn from participants' accounts disclosing their race. Finally, our analysis was limited to the 'results' and 'findings' sections of the selected articles; however, all sections of the articles were read to provide a deep understanding of the topic. We acknowledge that themes developed in this synthesis may be similar to findings reported in the included articles since it was not our intention to develop new interpretations or theories, and we wanted to stay close to the experiences reported by the participants in the included articles.

Conclusion

This review and thematic synthesis offers an overview of the experiences that affect the mental health and mental wellbeing of Black students studying at universities in the UK as reported in the available literature. Our results suggest that there are both personal and institutional factors that affect the mental health and mental wellbeing of Black students. Further research into how the learning, social, living, cultural and physical environment of universities affects the mental health of Black university students; and the differences in the lived experiences of Black university students across different identities and social statuses would give valuable insights. The impact of

For peer review only - http://bmjopen.bmj.com/site/about/guidelines.xhtml

institutional issues of racism and sexism on the mental health of Black students studying at UK higher education need to be further researched and explicitly addressed by policymakers and key decisionmakers within universities.

Twitter: @NkasiStoll

Acknowledgements: The authors would like to acknowledge Dionne Laporte for proof-reading the draft of this manuscript. NS carried out this work as part of a London Interdisciplinary Social Science Doctoral Training Partnership funded doctoral project.

Contributors: NS (lead author) conceived the study which was further refined by SH (joint senior author) and HL (joint senior author). NS developed the detailed methodology, undertook database searches and title and abstract review. Each study retained for full-text review was reviewed by NS. YY reviewed 50% of the full text. Discrepancies between NS and YY regarding inclusion or exclusion were resolved by SH. NS and YY made the critical appraisal of included studies. The thematic synthesis was conducted by NS and revised by YY, SH, HL. All authors contributed to the manuscript and approved the final version.

Funding: NS was supported by Economic and Social Research Council [grant number ES/P000703/1] via the London Interdisciplinary Social Science Doctoral Training Partnership.

SH is partly supported by the Economic and Social Research Council Centre for Society and Mental Health at King's College London [ES/S012567/1] and by the National Institute for Health Research (NIHR) Maudsley Biomedical Research Centre at South London and Maudsley NHS Foundation Trust and King's College London [BRC-1215-20018]. The views expressed are those of the authors and not necessarily those of the NHS, the NIHR or the Department of Health and Social Care, Wellcome Trust, ESRC or King's College London.

HL currently receives funding for successful grants as a PI or co- PI: Wellcome Trust Institutional Strategic Support Fund [N/A]; King's Health Partner Multiple Long-Term Conditions Challenge Fund [N/A]; National Axial Spondyloarthritis Society [N/A]; UKRCI Medical Research Council

[MR/S001255/1]; Medical Research Council [MR/R023697/1]; National Institute for Health Research [RP-PG-0610-10066]; Guy's and St. Thomas' Charity London [EFT151101]; and Versus Arthritis [N/A].

NB is partly supported by the Economic and Social Research Council funding for SMaRteN [ES/S00324X/1].

The funders had no involvement in the study design; collection, analysis and interpretation of the data; writing of the paper; or decision to submit the paper for publication.

Competing interests: None declared.

Patient and public involvement: The lead author is a Black Caribbean PhD student, and the second author is a Black African master's student. Both were involved in the design, conduct, reporting, and dissemination of this research.

Patient consent for publication: Not required.

Data availability statement: No data are available.

Figure Legend

Figure 1. PRISMA flow diagram

Table 1. Characteristics of included studies (in date order)

Table 2. Critical Appraisal Skills Programme (CASP) Checklist for included articles (in date order)

Table 3. Concept matrix for included studies (in date order)

References

For peer review only - http://bmjopen.bmj.com/site/about/guidelines.xhtml

1. Hubble, S. and Bolton, P. (2020). Support for students with mental health issues in higher education in England. Accessed from: https://commonslibrary.parliament.uk/
2. Office for Students (2019). Mental health: Are all students being properly supported? Accessed from: https://www.officeforstudents.org.uk/media/b3e6669e-5337-4caa-9553-049b3e8e7803/insight-brief-mental-health-are-all-students-being-properly-supported.pdf
3. Universities UK (2017). Higher Education in Facts and Figures. Accessed from: http://www.universitiesuk.ac.uk/facts-and-stats/data-and-analysis/Documents/higher-education-in-facts-and-figures-2017.pdf
4. Universities UK (2018). 'Minding our future: Starting a conversation about the support of student mental health'. Accessed from: https://www.universitiesuk. ac.uk/policy-and-analysis/reports/Pages/minding-ourfuture-starting-a-conversation-support-student-mentalhealth.aspx
5. Temple, P., Callender, C., Grove, L. & Kersh, N. (2016) Managing the student experience in English higher education: differing responses to market pressures. London Review of Education, 14 (1). pp. 33-46. DOI: 10.18546/LRE.14.1.05
6. Stallman, H. M. (2008). Prevalence of psychological distress in university students. Australian Family Physician, 37(8), 673– 677.
7. Friedlander, L., Reid, G., Shupak, N., & Cribbie, R. (2007). Social Support, Self-Esteem, and Stress as Predictors of Adjustment to University Among First-Year Undergraduates. Journal of College Student Development, 48(3) 259-274. https://doi.org/10.1353
8. Andrews, B. & Wilding J.M. (2004). The relation of depression and anxiety to life-stress and achievement in students. British Journal of Psychology, 95, 509-521.
9. Cooke, R., Bewick, B.M., Barkham, M., Bradley, M. & Audin, K. (2006). Measuring, monitoring and managing the psychological well-being of first year university students. British Journal of Guidance and Counselling, 34, 505-518
10. Chung, W. W., & Hudziak, J. J. (2017). The Transitional Age Brain: "The Best of Times and the Worst of Times". Child and adolescent psychiatric clinics of North America, 26(2), 157–175. https://doi.org/10.1016/j.chc.2016.12.017).
11. Kessler, R. C., Amminger, G. P., Aguilar-Gaxiola, S., Alonso, J., Lee, S., & Ustün, T. B. (2007). Age of onset of mental disorders: a review of recent literature. *Current opinion in psychiatry*, *20*(4), 359–364. https://doi.org/10.1097/YCO.0b013e32816ebc8c
12. Boden, J.M. & Foulds, J.A. (2016). Major depression and alcohol use disorder in adolescence: Does comorbidity lead to poorer outcomes of depression? Journal of Affective Disorders, 206, 287-293. https://doi.org/10.1016/j.jad.2016.09.004.

For peer review only - http://bmjopen.bmj.com/site/about/guidelines.xhtml

13. McGorry, P. D., Purcell, R., Goldstone, S., & Amminger, G. P. (2011). Age of onset and timing of treatment for mental and substance use disorders: implications for preventive intervention strategies and models of care. Current opinion in psychiatry, 24(4), 301–306. https://doi.org/10.1097/YCO.0b013e3283477a09
14. Hawton, K., Saunders, K. E., & O'Connor, R. C. (2012). Self-harm and suicide in adolescents. Lancet (London, England), 379(9834), 2373–2382. https://doi.org/10.1016/S0140-6736(12)60322-5).
15. Eskin, M., Voracek, M., Tran, T., Sun, J., Janghorbani, M., Giovanni Carta, M., … & Harlak, H. (2016). Suicidal Behavior and Psychological Distress in University Students: A 12-Nation Study. Archives of Suicide Research. Archives of Suicide Research, 20(3), 369-388. doi: 10.1080/13811118.2015.1054055
16. Higher Education Statistics Agency (2019). Students by level of study and academic year. Accessed from: https://www.hesa.ac.uk/data-and-analysis/students/chart-1
17. Neves, J. (2019). UK Engagement Survey 2019. Advance HE. Accessed from: https://www.advance-he.ac.uk/knowledge-hub/uk-engagement-survey-2019
18. Universities UK (2019). Black, Asian and Minority Ethnic student attainment at UK universities: #closingthegap. Acessed from: https://www.universitiesuk.ac.uk/policy-and-analysis/reports/Documents/2019/bame-student-attainment-uk-universities-closing-the-gap.pdf
19. Livesey, A (2019) BAME underrepresentation in UK universities: a view from the humanities. Innovations in Practice, 13 (2) 11-17 http://dx.doi.org/10.24377/LJMU.iip.vol13iss2artic...
20. Schucan Bird, K., Pitman, L. How diverse is your reading list? Exploring issues of representation and decolonisation in the UK. High Educ 79, 903–920 (2020). https://doi.org/10.1007/s10734-019-00446-9
21. Leading Routes. (2019). The broken pipeline: Barriers to Black PhD students accessing research council funding. UCL/Leading Routes
22. Smith, S. 2017. "Exploring the Black and Minority Ethnic (BME) Student Attainment Gap: What Did It Tell Us? Actions to Address Home BME Undergraduate Students' Degree Attainment." Journal of Perspectives in Applied Academic Practice 5: 48–57.)
23. Rollock, N. and Gillborn, D. (2011) Critical Race Theory (CRT), British Educational Research Association online resource. Available online at https://www.bera.ac.uk/publication/critical-race-theory-crt. Last accessed July 2020.
24. Tong, A., Flemming, K., McInnes, E., Oliver, S., & Craig, J. (2012). Enhancing transparency in reporting the synthesis of qualitative research: ENTREQ. *BMC medical research methodology*, *12*, 181. https://doi.org/10.1186/1471-2288-12-181

For peer review only - http://bmjopen.bmj.com/site/about/guidelines.xhtml

25. Morgan, R. L., Whaley, P., Thayer, K. A., & Schünemann, H. J. (2018). Identifying the PECO: A framework for formulating good questions to explore the association of environmental and other exposures with health outcomes. Environment international, 121(Pt 1), 1027–1031. https://doi.org/10.1016/j.envint.2018.07.015

26. Critical Appraisal Skills Programme (2018). CASP Qualitative Checklist. [online]. Accessed: https://casp-uk.net/wp-content/uploads/2018/01/CASP-Systematic-Review-Checklist_2018.pdf

27. Braun, V., & Clarke, V. (2006). Using thematic analysis in psychology. *Qualitative Research in Psychology, 3*(2), 77–101. https://doi.org/10.1191/1478088706qp063oa

28. Thomas, J., & Harden, A. (2008). Methods for the thematic synthesis of qualitative research in systematic reviews. *BMC medical research methodology*, *8*, 45. https://doi.org/10.1186/1471-2288-8-45

29. Moher D, Liberati A, Tetzlaff J, Altman DG, The PRISMA Group (2009). *P*referred *R*eporting *I*tems for *S*ystematic Reviews and *M*eta-*A*nalyses: The PRISMA Statement. PLoS Med 6(7): e1000097. doi:10.1371/journal.pmed1000097

30. Hayford, A. (2019). A Thematic Analysis Exploring BAME Students Perceptions of Accessing Support Services at University

31. Jones, C. (2017). Black and minority ethnic postgraduates and mental health.Accessed from: https://www.blogs.hss.ed.ac.uk/pubs-and-publications/2017/12/01/black-minority-ethnic-postgraduates-mental-health/

32. Alloh, F. T., Tait, D., & Taylor, C. (2018). Away from Home: A Qualitative Exploration of Health Experiences of Nigerian Students in a U.K. University. Journal of International Students, 8(1), 1–20. Social Science Premium Collection. https://doi.org/10.5281/zenodo.1101024

33. Arday, J. (2018). Understanding Mental Health: What Are the Issues for Black and Ethnic Minority Students at University? Social Sciences, 7(10). Social Science Premium Collection. https://doi.org/10.3390/socsci7100196

34. Jackson-Cole, D. (2019).Navigating Toward Success: Black and Minority Ethnic Students In Postgraduate Science, Technology, Engineering And Mathematics Courses In England. 317. Accessed from: https://repository.uel.ac.uk/download/0a1bf27b3ce062c7bc8fca4abf37529f82da68723e654815b29ba71b0b0ee117/2999353/2019_PhD_Jackson-Cole.pdf

35. Myrie, C. V. and Gannon, K. (2013). "Should I really be here?" Exploring the relationship between Black men's conceptions of wellbeing, subject positions and help-seeking behaviour. Diversity and Equality in Health and Care. 10 (1), pp. 1-22

For peer review only - http://bmjopen.bmj.com/site/about/guidelines.xhtml

36. Bunce, L., King, N., Saran, S., & Talib, N. (2019). Experiences of black and minority ethnic (BME) students in higher education: Applying self-determination theory to understand the BME attainment gap. Studies in Higher Education, 1–14. https://doi.org/10.1080/03075079.2019.1643305
37. Cummings, S. (2018). Attainment and identity in the face of dual oppression: Exploring the educational experiences of British females of Caribbean heritage. Accessed from: http://etheses.Whiterose.ac.uk/21340/
38. Akel, S. (2019). Insider-Outsider: The Role of Race in Shaping the Experiences of Black and Minority Ethnic Students. 58. Accessed from: https://www.gold.ac.uk/media/docs/reports/Insider-Outsider-Report-191008.pdf
39. Bhatti, S. (2019). A Mixed Methods Study Investigating Black British Women's Perceptions and Attitudes Of Body Image And Body Appreciation. 214. Acessed from: https://openaccess.city.ac.uk/id/eprint/23922/
40. Crittle, K. (2018). Adjusting to UK life as an African American student. University Wire. Social Science Premium Collection. https://search.proquest.com/docview/2139161092?accountid=11862
41. Baxe, K. (2018). NUS Black Students Conference 2018. Retrieved 14 July 2020, from: https://www.centralsu.com/single-post/2018/05/15/NUS-Black-Students-Conference-2018
42. Phillips, C. (2011) *Institutional racism and ethnic inequalities: an expanded multilevel framework. Journal of social policy*, 40 (01). pp. 173-192.
43. Arday, J., & Mirza, H. (Eds.). (2018). *Dismantling race in higher education*. Basingstoke: Palgrave.
44. Warmington, P. (2020). Critical race theory in England: impact and opposition, Identities, 27:1, 20-37, Doi.: 10.1080/1070289X.2019.1587907
45. Bhopal, K. & Pitkin, C. (2020) 'Same old story, just a different policy': race and policy making in higher education in the UK, Race Ethnicity and Education, 23:4, 530-547, DOI: 10.1080/13613324.2020.1718082
46. Doharty, N., Madriaga, M., & Joseph-Salisbury, R. (2020): The university went to 'decolonise' and all they brought back was lousy diversity double-speak! Critical race counter-stories from faculty of colour in 'decolonial' times, Educational Philosophy and Theory, DOI: 10.1080/00131857.2020.176960

For peer review only - http://bmjopen.bmj.com/site/about/guidelines.xhtml

47. Ledesma, M. C., & Calderón, D. (2015). Critical Race Theory in Education: A Review of Past Literature and a Look to the Future. *Qualitative Inquiry*, *21*(3), 206–222. https://doi.org/10.1177/1077800414557825
48. Chakrabarty, N., Roberts, L. & Preston, J. (2012). Critical Race Theory in England, Race Ethnicity and Education, 15:1, 1-3, Doi: 10.1080/13613324.2012.638860
49. Mirza, H. S. (2018). Racism in higher education: What then, can be done? In J. Arday, & H. S. Mirza (Eds.), Dismantling race in higher education: Racism, Whiteness and decolonising the academy (pp. 1–7). Palgrave Macmillan
50. Gunnell, D., Caul, S., Appleby, L., John, A., & Hawton, K. (2020). The incidence of suicide in University students in England and Wales 2000/2001–2016/2017: Record linkage study. Journal of Affective Disorders, 261, 113-120. https://doi.org/10.1016/j.jad.2019.09.079
51. Times Higher Education (2020). We are at a crucial moment to address racial justice in higher education. Accessed from: https://www.timeshighereducation.com/letters/we-are-crucial-moment-address-racial-justice-higher-education
52. BBC News (2020). Aberdeen University students detail racism experience in letter. Accessed from: https://www.bbc.co.uk/news/uk-scotland-north-east-orkney-shetland-53106494
53. Oxford SU (2020). Open Letter to the Vice Chancellor on structural anti-Blackness at Oxford University. Accessed from: https://www.oxfordsu.org/news/article/6013/Open-Letter-to-the-Vice-Chancellor-on-structural-anti-Blackness-at-Oxford-University/
54. Jones, A. (2020). Open letter to Adam Tickell addressing racial inequality. Accessed from: https://thetab.com/uk/sussex/2020/06/04/open-letter-to-adam-tickell-addressing-racial-inequality-41924
55. Crawley, S. (2020). Open letters demand University does more to tackle racism. Accessed from: https://www.varsity.co.uk/news/19541
56. Taylor, Y. (2020). Over 2,200 sign open letter to St Andrews Principal demanding action over diversity policies. Accessed from: https://thetab.com/uk/stand/2020/06/10/over-2200-sign-open-letter-to-st-andrews-principal-demanding-action-over-diversity-policies-22866
57. University of Portsmouth (2020). Scholarship and Support Fund for Black Students. Accessed from: https://www.port.ac.uk/about-us/alumni/donate/support-black-students

For peer review only - http://bmjopen.bmj.com/site/about/guidelines.xhtml

Figure 1 PRISMA flow diagram

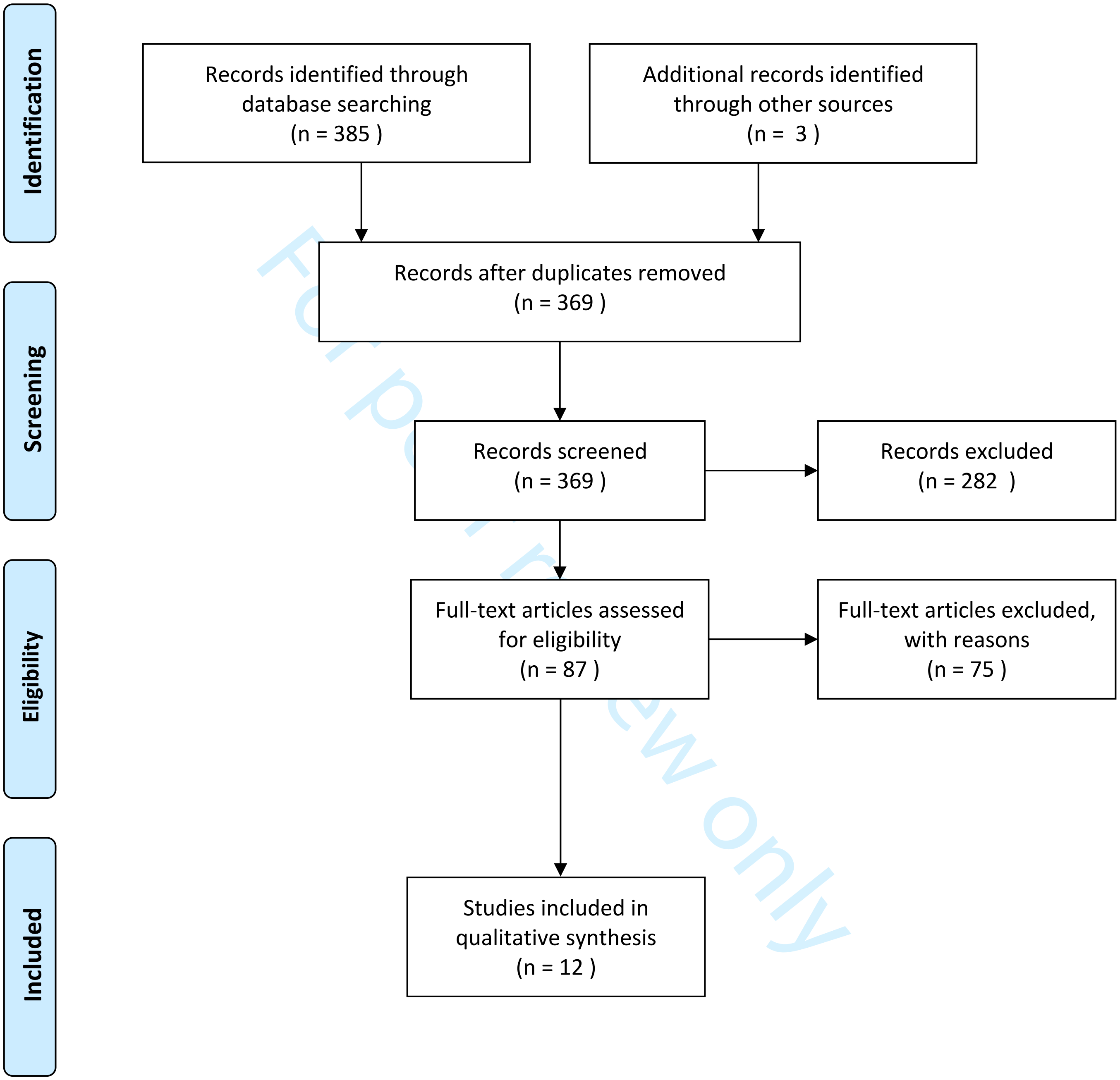

PRISMA 2009 Checklist

Section/topic	#	Checklist item	Reported on page #
**TITLE**			
Title	1	Identify the report as a systematic review, meta-analysis, or both.	1
**ABSTRACT**			
Structured summary	2	Provide a structured summary including, as applicable: background; objectives; data sources; study eligibility criteria, participants, and interventions; study appraisal and synthesis methods; results; limitations; conclusions and implications of key findings; systematic review registration number.	2-3
**INTRODUCTION**			
Rationale	3	Describe the rationale for the review in the context of what is already known.	4-5
Objectives	4	Provide an explicit statement of questions being addressed with reference to participants, interventions, comparisons, outcomes, and study design (PICOS).	5
**METHODS**			
Protocol and registration	5	Indicate if a review protocol exists, if and where it can be accessed (e.g., Web address), and, if available, provide registration information including registration number.	N/A
n	6	Specify study characteristics (e.g., PICOS, length of follow-up) and report characteristics (e.g., years considered, language, publication status) used as criteria for eligibility, giving rationale.	5-6
Information sources	7	Describe all information sources (e.g., databases with dates of coverage, contact with study authors to identify additional studies) in the search and date last searched.	5
Search	8	Present full electronic search strategy for at least one database, including any limits used, such that it could be repeated.	5-6
Study selection	9	State the process for selecting studies (i.e., screening, eligibility, included in systematic review, and, if applicable, included in the meta-analysis).	6
Data collection process	10	Describe method of data extraction from reports (e.g., piloted forms, independently, in duplicate) and any processes for obtaining and confirming data from investigators.	6
Data items	11	List and define all variables for which data were sought (e.g., PICOS, funding sources) and any assumptions and simplifications made.	5-6 (Constructs not variables)
Risk of bias in individual studies	12	Describe methods used for assessing risk of bias of individual studies (including specification of whether this was done at the study or outcome level), and how this information is to be used in any data synthesis.	6
Summary measures	13	State the principal summary measures (e.g., risk ratio, difference in means).	N/A

For peer review only

PRISMA 2009 Checklist

Synthesis of results	14	Describe the methods of handling data and combining results of studies, if done, including measures of consistency (e.g., I^2) for each meta-analysis.	N/A – qual thematic synthesis approach

Section/topic	#	Checklist item	Reported on page #
Risk of bias across studies	15	Specify any assessment of risk of bias that may affect the cumulative evidence (e.g., publication bias, selective reporting within studies).	N/A
Additional analyses	16	Describe methods of additional analyses (e.g., sensitivity or subgroup analyses, meta-regression), if done, indicating which were pre-specified.	N/A
**RESULTS**			
Study selection	17	Give numbers of studies screened, assessed for eligibility, and included in the review, with reasons for exclusions at each stage, ideally with a flow diagram.	7<br>Supplementary file #1
Study characteristics	18	For each study, present characteristics for which data were extracted (e.g., study size, PICOS, follow-up period) and provide the citations.	7-8
Risk of bias within studies	19	Present data on risk of bias of each study and, if available, any outcome level assessment (see item 12).	9-10
Results of individual studies	20	For all outcomes considered (benefits or harms), present, for each study: (a) simple summary data for each intervention group (b) effect estimates and confidence intervals, ideally with a forest plot.	10-11 (concept matrix)
Synthesis of results	21	Present results of each meta-analysis done, including confidence intervals and measures of consistency.	11-15
Risk of bias across studies	22	Present results of any assessment of risk of bias across studies (see Item 15).	N/A
Additional analysis	23	Give results of additional analyses, if done (e.g., sensitivity or subgroup analyses, meta-regression [see Item 16]).	N/A
**DISCUSSION**			
Summary of evidence	24	Summarize the main findings including the strength of evidence for each main outcome; consider their relevance to key groups (e.g., healthcare providers, users, and policy makers).	16
Limitations	25	Discuss limitations at study and outcome level (e.g., risk of bias), and at review-level (e.g., incomplete retrieval of identified research, reporting bias).	17-18
Conclusions	26	Provide a general interpretation of the results in the context of other evidence, and implications for future research.	18-19
**FUNDING**			

For peer review only

PRISMA 2009 Checklist

Funding	27	Describe sources of funding for the systematic review and other support (e.g., supply of data); role of funders for the systematic review.	19-20

From: Moher D, Liberati A, Tetzlaff J, Altman DG, The PRISMA Group (2009). Preferred Reporting Items for Systematic Reviews and Meta-Analyses: The PRISMA Statement. PLoS Med 6(7): e1000097. doi:10.1371/journal.pmed1000097

For more information, visit: **www.prisma-statement.org**.

For peer review only

For peer review only - http://bmjopen.bmj.com/site/about/guidelines.xhtml

BMJ Open

Mental Health and Mental Wellbeing of Black Students at UK Universities: A Review and Thematic Synthesis

Journal:	*BMJ Open*
Manuscript ID	bmjopen-2021-050720.R1
Article Type:	Original research
Date Submitted by the Author:	15-Oct-2021
Complete List of Authors:	Stoll, Nkasi; King's College London Institute of Psychiatry Psychology and Neuroscience, Department of Psychological Medicine<br>Yalipende, Yannick; University College London, Division of Psychology and Language Sciences<br>Byrom, Nicola; King's College London Institute of Psychiatry Psychology and Neuroscience, Department of Psychology<br>HATCH, STEPHANI; King's College London Institute of Psychiatry Psychology and Neuroscience, Department of Psychological Medicine; King's College London, ESRC Centre for Society and Mental Health<br>Lempp, Heidi; King's College London Faculty of Life Sciences and Medicine, Department of Inflammation Biology
**Primary Subject Heading**:	Mental health
Secondary Subject Heading:	Mental health, Qualitative research, Sociology, Research methods, Health policy
Keywords:	MENTAL HEALTH, QUALITATIVE RESEARCH, PSYCHIATRY, PUBLIC HEALTH, SOCIAL MEDICINE, EPIDEMIOLOGY

SCHOLARONE™ Manuscripts

I, the Submitting Author has the right to grant and does grant on behalf of all authors of the Work (as defined in the below author licence), an exclusive licence and/or a non-exclusive licence for contributions from authors who are: i) UK Crown employees; ii) where BMJ has agreed a CC-BY licence shall apply, and/or iii) in accordance with the terms applicable for US Federal Government officers or employees acting as part of their official duties; on a worldwide, perpetual, irrevocable, royalty-free basis to BMJ Publishing Group Ltd ("BMJ") its licensees and where the relevant Journal is co-owned by BMJ to the co-owners of the Journal, to publish the Work in this journal and any other BMJ products and to exploit all rights, as set out in our licence.

The Submitting Author accepts and understands that any supply made under these terms is made by BMJ to the Submitting Author unless you are acting as an employee on behalf of your employer or a postgraduate student of an affiliated institution which is paying any applicable article publishing charge ("APC") for Open Access articles. Where the Submitting Author wishes to make the Work available on an Open Access basis (and intends to pay the relevant APC), the terms of reuse of such Open Access shall be governed by a Creative Commons licence – details of these licences and which Creative Commons licence will apply to this Work are set out in our licence referred to above.

Other than as permitted in any relevant BMJ Author's Self Archiving Policies, I confirm this Work has not been accepted for publication elsewhere, is not being considered for publication elsewhere and does not duplicate material already published. I confirm all authors consent to publication of this Work and authorise the granting of this licence.

For peer review only

Mental Health and Mental Wellbeing of Black Students at UK Universities: A Review and Thematic Synthesis

Ms Nkasi Stoll*; nkasi.1.stoll@kcl.ac.uk; King's College London, Institute of Psychiatry Psychology and Neuroscience, Department of Psychological Medicine, London, UK

E.319, Institute of Psychiatry Psychology and Neuroscience, Department of Psychological Medicine, Camberwell, London, UK, SE5 8AF

Mr Yannick Yalipende; yannick.yalipende.20@ucl.ac.uk; University College London, Division of Psychology and Language Sciences, London, UK

Dr Nicola Bryom; Nicola.byrom@kcl.ac.uk; King's College London, Institute of Psychiatry Psychology and Neuroscience, Department of Psychology, London, UK

Professor Stephani Hatch*; stephani.hatch@kcl.ac.uk; King's College London, Institute of Psychiatry Psychology and Neuroscience, Department of Psychological Medicine, London, UK; King's College London, ESRC Centre for Society and Mental Health, London, UK

Dr Heidi Lempp*; heidi.lempp@kcl.ac.uk; King's College London, Faculty of Life Sciences and Medicine, Department of Inflammation Biology, Centre f or Rheumatic Diseases, London, UK

Five keyword(s): MENTAL HEALTH**;** QUALITATIVE RESEARCH**;** PSYCHIATRY**;** PUBLIC HEALTH**;** SOCIAL MEDICINE**;** EPIDEMIOLOGY

Word count: 3856

For peer review only - http://bmjopen.bmj.com/site/about/guidelines.xhtml

Abstract

Background: There is a knowledge gap about the experiences that affect the mental health of Black university students in the UK. Current research is focused on understanding the continuation, attainment and progression gap between Black students and non-Black students. It is essential to know more about the interactions between personal and institutional factors on the mental health of Black students to explain the inequalities in their experiences and outcomes across the university lifecycle. The current study set out to thematically synthesise articles that explore the experiences that affect the mental health and mental wellbeing of Black university students in the UK.

Methods: This study is a qualitative thematic synthesis of a literature review. We developed search strategies for four online databases (PubMed, Social Science Premium Collection via ProQuest, Open Access Theses and Dissertations, and Open Grey) covering January 2010 to July 2020. This search was combined with a manual search of reference lists and related citations. All articles in English addressing mental health and mental wellbeing experiences among Black university students studying at a UK university were included. Critical Appraisal Skills Programme (CASP) Checklist was used to assess bias. A thematic synthesis was conducted to using Braun and Clarke (2006)'s six step guide to develop descriptive themes and analytical constructs.

Results: Twelve articles were included. Several themes were identified as affecting the mental health of Black university students in the UK: academic pressure, learning environment, Black gendered experience, isolation and alienation, culture shock, racism and support.

Discussion: This review provides an appraisal of the factors affecting the mental health and mental wellbeing of Black students at UK universities, which need to be addressed by Higher Education policymakers and key decision-makers. Further research is needed about the mental health experiences of Black university students in relation to Black identities, suicidality, mental health language, the physical environment, and racism and other institutional factors.

For peer review only

Strengths and Limitations of this study

- This is the first review and thematic synthesis to provide an overview of the personal and institutional experiences that affect the mental health and mental wellbeing of Black students studying at UK universities
- A thematic synthesis approach was used to describe and compare the main findings, to help inform policy and interventions
- Database and manual searching were extensive, screening 369 articles.
- It is possible not all relevant articles were found due to inconsistent terminology for the racial category "Black" and "Mixed" in existing studies.

For peer review only

Introduction

There is increasing concern about the mental health and mental wellbeing of university students in the United Kingdom (UK)[1-4]. The university student lifecycle has been conceptualised in terms of (i) the application experience which covers the interactions between potential students and the institution up to the point of arrival; (ii) the academic experience which encompasses students' interactions with their institution of study; (iii) the campus experience, which includes students' life not directly connected with study and may include activities away from the campus; and (iv) the graduate experience which covers the institution's role in assisting students' transition to employment or further study[5].

Throughout the lifecycle, British and international students are exposed to a range of experiences that make universities a high-risk period for maladaptive coping and the possible onset of poor mental health. These experiences include (but are not limited to): individuation, separation from family, new social connections, increased autonomy and responsibility, academic-related stress, financial concerns, sleep disruption, balancing conflicting demands of studying with personal and family life, and exposure to risky behaviours, including recreational drug use and alcohol binging[6-10].

Late adolescence through young adulthood is a peak age period for the first onset of mental health problems[11], which overlaps the period that many young adults enter and navigate the university student lifecycle. Unsupported mental health problems are associated with progression to other co-morbid disorders, substance use disorders, self-harm, and suicide ideation, and attempts[12-15]. According to Higher Education Statistics Agency, there are 2.4 million university students in higher education in the UK[16]; which means that universities are in the best position to provide prevention and intervention to many young adults during a critical transitional period.

In the UK, Black students are more likely than White students to self-report high levels of engagement and participation in their university studies; covering seven categories of engagement including course challenge, critical thinking, and research and inquiry [17]. Despite high engagement

For peer review only - http://bmjopen.bmj.com/site/about/guidelines.xhtml

levels, Black students report lower satisfaction and are less likely to complete their course, achieve a good degree (1st or 2:1), and progress to further education[18]. In addition to the aforementioned experiences and risk factors for poor mental health, there is tentative research around the institutional issues affecting Black university student mental health including inequality in access, under-representation, the attainment gap, institutional racism, and de-colonising the curriculum[19-23]. To explain the inequalities in student experiences and outcomes across the university lifecycle, further understanding of the interactions between personal and institutional factors on the mental health of Black students' is needed.

This review aims to synthesise existing studies that explore mental health and mental wellbeing in Black students at UK universities. Therefore, our research question is as follows: what are the experiences that affect the mental health and mental wellbeing of Black university students in the UK, as reported in the literature?

Methods

Design

A review of the literature including quantitative, qualitative, and mixed studies was conducted on peer-reviewed and non-peer-reviewed articles, addressing mental health and mental wellbeing of Black students at UK universities. Reporting of this review was guided by the Enhancing Transparency of Reporting the Synthesis of Qualitative Research framework [24], as the included articles were all qualitative.

Search methods

The following databases were searched for the period of January 2010 to July 2020: PubMed, Social Science Premium Collection via ProQuest, Open Access Theses and Dissertations, and Open Grey. The search strategy included several combinations of the following medical subject heading (MeSH) terms and keywords[25]: "mental health" or "psychological distress" or "mental

For peer review only - http://bmjopen.bmj.com/site/about/guidelines.xhtml

wellbeing" and "University student" and "Black Caribbean" or "Black African" or "Black Mixed" or "Black ethnic*".

Data collection

All search results were saved to Zotero bibliographic management software and duplicates were removed. To be included in the review, articles had to (1) focus on the mental health or mental wellbeing of Black students at UK universities (2) apply empirical (e.g., quantitative, qualitative, or mixed-method research) or non-empirical research methods (e.g., theoretical, opinion-based, student transcripts, policy papers) (3) be written in English, (4) be conducted in the UK. The first author (NS) removed all articles that did not fit the inclusion criteria after full-text review. NS manually searched reference lists of eligible articles and contacted lead authors to search for unpublished literature. The authors decided to be lenient in the inclusion criteria during the screening and inclusion of articles because existing research on Black university student mental health is limited, and to enable the inclusion of articles that add value to the discussion of the topic. All included articles were critically appraised by NS and the author YY using the 'Critical Appraisal Skills Programme' (2018)[26] checklist for qualitative research.

Data analysis and synthesis

For each article, all text from 'Results/Findings' and 'Discussion' were extracted and imported into NVivo V.12 software (NVivo Qualitative data analysis Software; QSR International, V.12, 2018). Study characteristics were extracted into a Microsoft Excel grid by NS and revised by YY. Full-text screening, selection, data extraction, and critical appraisal of all articles was conducted prior to data analysis. A thematic analysis was conducted to describe and compare the main findings, following Braun and Clarke's (2006)[27] six-step guide. NS carefully read each article to familiarise themselves with the content, annotating initial ideas for codes and themes. NS then read and re-read the articles to develop an initial coding scheme, using a constant comparative approach[28]. Themes and sub-themes were identified to capture important patterns across and within the articles included. Six articles (50% of the sample) were randomly selected to be coded independently by YY, to provide validation. The codes were examined for similarities and

For peer review only - http://bmjopen.bmj.com/site/about/guidelines.xhtml

differences and then organised into a hierarchy to create the final codes. Any differences were resolved by SH. The final codes were validated by NS, YY, SH, and HL, before being synthesized into the final thematic framework presented in this paper to minimise researcher subjectivity and improve the credibility of the work.

Patient and Public Involvement

The lead author is a Black Caribbean PhD student, and the second author is a Black African master's student. Both were involved in the design, conduct, reporting, and dissemination of this research.

Results

Included studies

Figure 1 shows the number of articles identified and rejected at each stage of the review process[29]. The search strategies identified 388 references of which 369 were kept after removing duplicates. After screening, 87 articles were read completely. Of these, 75 articles were excluded. A final sample of n=12 studies were included. Table 1 presents the characteristics of the included studies.

Table 1 Characteristics of included studies

**Author(s)**	**Database**	**Type of article**	**No. of Black participants**	**Subject Characteristics**	**Location**	**Psychological phenomena explored**	**Methods**
Hayford, A. (2019)[30]	Hand searched	Dissertation	6	One was Caribbean, one was Somalian, one was Nigerian, two were mixed black African, and one was Ghanaian	East Anglia	Wellbeing	Interviews
Jones, C. (2017)[31]	PubMed	Blog	1	Black academic staff	UK	Wellbeing	Personal reflections and notes
Alloh, F. T., Tait, D., &	Proquest	Journal article	9	International Nigerian students studying at the	southeast of England	Wellbeing	Interviews

For peer review only - http://bmjopen.bmj.com/site/about/guidelines.xhtml

Taylor, C. (2018)[32]				master's-level in a U.K. university			
Arday, J. (2018)[33]	Proquest	Journal article	14	32 BME university students	UK	Mental Health	Interviews focus Groups
Jackson-Cole, D. (2019)[34]	Open Grey	Doctoral thesis	10	Postgraduate students. two Black or Black British –Caribbean participants, seven Black or Black British –African, one Mixed –White and Black African	England	Wellbeing	Interviews
Myrie, C. V. and Gannon, K. (2013)[35]	Hand searched	Journal article	3	2 Black African Stud ents, 1 Black African and Caribbean Student	East and North London	Wellbeing	Interviews
Bunce, L., King, N., Saran, S., & Talib, N. (2019)[36]	PubMed	Journal article	14	14 full-time students from two degree programmes in health and social care related subjects - Black African (12) and White and Black Caribbean (2)	Unknown	Wellbeing	Focus Groups
Cummings, S. (2018)[37]	Open Grey	Doctoral thesis	5	Black women with Caribbean heritage, who were being educated at a university in the East Midlands	East Midlands	Wellbeing	Focus Groups
Akel, S. (2019)[38]	PubMed	Report	Not specified	195 BME students. 'Black' refers to people of African heritage and the diaspora.	London	Wellbeing	Interviews focus Groups
Bhatti, S. (2019)[39]	Open Grey	Doctoral thesis	5	Black students	London	Body image	Focus Groups
Crittle, K. (2018)[40]	Proquest	Blog	1	African American student	UK	Stress	Personal Reflections And Notes

For peer review only - http://bmjopen.bmj.com/site/about/guidelines.xhtml

Baxe, K. (2018)[41]	PubMed	Conference notes	1	Black students	UK	Mental Health	Personal Reflections And Notes

All included articles were assessed by NS and YY as reasonable quality and were therefore judged to have relevant contributions for the thematic synthesis (Table 2).

Table 2 Critical Appraisal Skills Programme (CASP) Checklist for included articles

	Was there a clear statement of the aims of the research?	Is a qualitative methodology appropriate?	Was the research design appropriate to address the aims of the research?	Was the recruitment strategy appropriate to the aims of the research?	Was the data collected in a way that addressed the research issue?	Has the relationship between researcher and participants been adequately considered?	Have ethical issues been taken into consideration?	Was the data analysis sufficiently rigorous?	Is there a clear statement of findings?	How valuable is the research?
Hayford, A. (2019)[30]	Yes	Yes	Yes	Yes	Yes	Yes	Yes	Yes	Yes	Valuable
Jones, C. (2017)[31]	No	N/A	N/A	N/A	N/A	N/A	N/A	N/A	Yes	Valuable
Alloh, F. T., Tait, D., & Taylor, C. (2018)[32]	Yes	Yes	Yes	Yes	Yes	No	Yes	Yes	Yes	Valuable
Arday, J. (2018)[33]	Yes	Yes	Yes	Yes	Yes	No	Can't Tell	Yes	Yes	Valuable
Jackson-Cole, D. (2019)[34]	Yes	Yes	Yes	Yes	Yes	Yes	Yes	Yes	Yes	Valuable
Myrie, C. V. and Gannon, K. (2013)[35]	Yes	Yes	Yes	Yes	Yes	No	Yes	Yes	Yes	Valuable
Bunce, L., King, N.,	Yes	Yes	Yes	Yes	Yes	No	Can't Tell	Yes	Yes	Valuable

For peer review only - http://bmjopen.bmj.com/site/about/guidelines.xhtml

Saran, S., & Talib, N. (2019)[36]										
Cummings, S. (2018)[37]	Yes	Yes	Yes	Yes	Yes	Yes	Yes	Yes	Yes	Valuable
Akel, S. (2019)[38]	Yes	Yes	Yes	Yes	Yes	No	Yes	Yes	Yes	Valuable
Bhatti, S. (2019)[39]	Yes	Yes	Yes	Yes	Yes	Yes	Yes	Yes	Yes	Valuable
Crittle, K. (2018)[40]	Yes	Yes	Yes	Yes	Yes	Yes	Yes	Yes	Yes	Valuable
Baxe, K. (2018)[41]	No	N/A	N/A	N/A	N/A	N/A	N/A	N/A	Yes	Valuable

Synthesised findings

The concept matrix for the findings can be seen in Table 3. We interpreted seven analytic themes derived from the participants' beliefs, perceptions, and experiences, and the article's authors' interpretations. The references for articles contributing to each of the themes are provided.

Table 3 Concept matrix for included studies

	**Support**	**Isolation and Alienation**	**Racism**	**Culture Shock**	**Black gendered experience**	**Learning environment**	**Academic pressure**
Hayford, A. (2019)[30]	X	X		X			
Jones, C. (2017)[31]	X		X	X			X
Alloh, F. T., Tait, D., & Taylor, C. (2018)[32]	X		X	X			X
Arday, J. (2018)[33]	X	X	X		X		X
Jackson-Cole, D. (2019)[34]	X		X		X	X	
Myrie, C. V. and Gannon, K. (2013)[35]	X	X			X		

For peer review only
For peer review only - http://bmjopen.bmj.com/site/about/guidelines.xhtml

Bunce, L., King, N., Saran, S., & Talib, N. (2019)[36]		X		X		X	
Cummings, S. (2018)[37]	X	X	X		X	X	
Akel, S. (2019)[38]		X	X			X	
Bhatti, S. (2019)[39]	X				X		
Crittle, K. (2018)[40]	X	X					
Baxe, K. (2018)[41]		X					

Academic pressure

In six out of 12 articles, academic pressure was a determinant of mental distress for Black students[30-35]. Students described their mental health as being adversely affected by the multiple and simultaneous academic tasks they were expected to complete during their degree[30-31]. Cumulatively, Black students reported their observation that, unlike their non-Black peers, they faced pressures from their families and the wider Black community which meant they needed to work harder to succeed at university; which further exacerbated mental distress[30,31,33,34]. This pressure came from their families continuously reminding them of the sacrifices and investments made, specifically immigration and financial strain[30,31,35]. Black students with pre-existing mental and physical health conditions have been reported to be especially vulnerable to poor mental health caused by this academic pressure[31-32]. There was some evidence that Black students might not seek mental health support and instead prioritise their continuation and success in academia, at the expense of their mental health, as a mental illness diagnosis might affect their success in academia[30,33].

Learning environment

In four of the 12 articles, researchers described Black students' perceptions of the university learning and teaching environment and how it impacted their mental health and mental wellbeing[34,

For peer review only - http://bmjopen.bmj.com/site/about/guidelines.xhtml

[36-38]. Lectures that had discussions on diversity, inclusion, ethnicity, race, and identity were said to be cathartic and liberating for Black students, which contributed to positive wellbeing[36-38]. Black students reported they had to censor themselves in academic spaces to be seen by White students and staff as acceptable and agreeable instead of loud, disruptive, and confrontational[37]. This behaviour was believed to improve their learning experience and relationship with teaching staff, at the expense of their wellbeing. Participants spoke about their lack of relatedness to the White majority academic staff, students, and teaching materials and racism in the classroom made them feel excluded, frustrated, distressed, discouraged, and unmotivated to engage in their degree course[34,36-38]. In university seminars, Black people were racially stereotyped as bad people, criminals, with lower intellectual ability by White students, and these stereotypes were sometimes reinforced by course materials[36-38]. Black female-identifying students recounted their academic knowledge was over-policed and over-scrutinised by teaching staff, inaccessibility of learning and wellbeing support compared to men and White women on their course, and the impact this had on their mental health and learning experience[34].

Black gendered experience

The Black student mental health experience of being at the intersection between race and the oppressive structures of traditional masculinities and femininities was discussed in five articles[33-35,37,39]. Black male-identifying students reported being affected by the discourses of hyper-masculinity, which put pressure on them to not show their emotions, cope with their mental distress and continue with their studies on their own[33,35]. This caused them to feel shame when seeking mental health support. For some female-identifying Black students, feelings about their health, ideal body shape, and size were influenced by the beliefs, culture, race, and ethnicity of the people in their lives[39]. The importance of seeing their body shape among Black students who looked like them was discussed. Some students felt the White body ideal is not the same as the Black body ideal, therefore visiting their country of origin positively impacted their stress levels, self-esteem, and body image. Social media and the music industry were mentioned as vehicles in which the ideal Black female body is narrated, scrutinised, and picked apart[39].

Isolation and Alienation

For peer review only - http://bmjopen.bmj.com/site/about/guidelines.xhtml

Eight out of 12 included studies discussed isolation and alienation[30,33,35-38,40-41]. Black students who struggled with their mental health experienced stigma from the Black community before attending university, leaving them isolated from their communities[30]. At university, participants reported being ignored or avoided by their non-Black peers in learning, social, and living environments which led to feelings of lack of belonging, diminished their overall wellbeing, and evoked a range of negative emotions, including discomfort, distress, frustration, and anger[30,33,36-38,40-41]. Those with little or no mental health support from family felt isolated and alienated from their friends, family, peers, and academic staff, and used denial to cope with the lack of support, which exacerbated their mental health problems[33].

Black students reported that a lack of relatedness to White students affected their wellbeing, undermined their motivation for academic success, and had a negative influence on their sense of autonomy and competence[30,36-38]. Black students mentioned White students did not understand that they have to reconstruct their identity in the White image and practice silence to be successful at university and that this process negatively affected their mental health[30].

Participants in one article discussed that academic staff do not always create safe and protective learning and living spaces for Black university students[38]. As a result, Black students reported they had to defend themselves, which made White students uncomfortable, leaving Black students feeling bullied, alienated, and alone. Participants who had the support of other Black students in their learning environment at university reported being more comfortable, understood, and happy[36]. However, this may not apply to Black students who are international students. One article reported a student being in tears, feeling alone and isolated, and almost dropping out of university due to their African accent isolating them from Black British students and non-Black students[36].

Culture Shock

There was evidence suggesting that Black international-status students had to adjust to new environments, cultures, and lifestyles in the UK which was characterised by changes in diet, weight, physical activity, alcohol, and smoking consumption, and led to stress, depression,

For peer review only - http://bmjopen.bmj.com/site/about/guidelines.xhtml

loneliness, and loss of confidence[30-32,36]. Black students who emigrated from a collectivist culture to the UK experienced culture shock that was emotionally stressful[32]. Contrastingly, there was evidence[32] to suggest Black home-status students had slightly poorer mental health and lower self-esteem than Black international-status students. Indeed, one article reported some Black international-status students had higher stress levels in their home country which gave them the perspective of living in the UK as being less stressful[32].

Racism

Five of the 12 included studies explored how institutional racism, discrimination, and hegemonic White privilege made universities toxic spaces for Black students, which affected their mental health and wellbeing[31, 33-34,37-38]. The constant stress of being confronted with racism (including racial microaggressions), discrimination, and having to survive hostile racist environments at university led to poor mental health and mental illness in Black students[31,33-34,37]. Racism had negative consequences on Black students' sense of belonging at university, motivation to socialise, their interactions with White students and staff, and their academic achievements and progression, which led to further mental distress. There is evidence to suggest Black students' recognition of racial difference had a greater impact on their mental wellbeing and potential or experienced mental health difficulties, than evidence that others treated them differently on account of their race[37]. Participants felt unable to express their feelings of racism and difference from White peers to others for fear they would not be understood, would be judged or rejected by peers and university staff, or due to uncertainty that these feelings were valid or legitimate[37]. These unarticulated feelings resulted in disengagement from university study and services.

Support

The aforementioned factors that possibly influence the mental health of Black students were discussed to affect and be affected by the type, access to, experience, and perceptions of mental health support[30-35, 37-40].

For peer review only - http://bmjopen.bmj.com/site/about/guidelines.xhtml

Black students reported they had to take care of their mental health by trying to remain positive and use distraction, research psycho-educational resources, change daily habits including exercise and nutrition, and use food to cope with stress at university and connect them to family[32-34, 37, 39].

Access to mental health support from family, friends, and peers was identified as a contributory factor to positive wellbeing and success at university[30-31,33-34,37,40]. One article reported Black students who did not have familial or social capital found accessing university mental health support more difficult[33]. Four included articles[31,33,35,40] had findings linked to mental health stigma and taboo leaving Black students feeling unsupported by their family at university and unable to get the professional support needed. Black-only spaces at university were reported as important to obtain support for mental and wellbeing[30-31,33,37,40]. These spaces gave students comfort, where they were not judged and misunderstood by non-Black students and staff; and could be with students from a shared background and experience. These Black-only spaces were especially important when there was a lack of Black students in classroom spaces and could be online or in person. Black students also placed importance on mental health support from an understanding religious organisation or chaplain at their university[30-31,33].

Included articles described Black students' beliefs that university services were institutionally racist, treated Black students as a monolithic group, thereby failed to provide appropriate services; and did not take responsibility for this[30-31,33,38]. Participants' awareness that White mental health practitioners at university services were the majority race prevented them from accessing services, because of perceived racial stereotyping and lack of trust[33,38]. Some Black students avoided university mental health services to such an extent that they were not knowledgeable about what was available for them[30]. Black students who needed mental health support did not seek such services until in crisis[30,33] and when they did, faced long waiting times[33]. Black students who did receive help from university mental health services reported experiencing racialised stereotypes that affected their relationship with White mental health practitioners[30-31,33,38]. They had to educate practitioners on Black culture and the Black experience (which included how racism and micro-aggressions affected their mental health), which was a burden on their mental health and wellbeing[30-31,33,38]. Because of this, students felt more comfortable with a Black, Asian or other minoritised ethnic mental health practitioner[30,33,38]. Black students described a sense of

For peer review only - http://bmjopen.bmj.com/site/about/guidelines.xhtml

helplessness regarding their mental healthcare treatment[30,33,38]. One article discussed Black students felt unable to challenge mental health professionals about their healthcare treatment because they may be labelled as difficult or unstable, and worsen their relationship with the mental healthcare provider[33].

Discussion

This review and thematic synthesis provides evidence on how the following experiences affect the mental health and mental wellbeing of Black university students in the UK: academic pressure, learning environment, Black gendered experience, isolation and alienation, culture shock, racism, and support.

The study findings highlight that for Black university students their mental health and mental wellbeing may be deeply rooted in institutional factors or dimensions, largely racism[31,33-34,37-39], which negatively affects most aspects of their higher education experiences, particularly in the learning, social, and living environments. Critical Race Theory in Education (CRT-E) is used to engage with and work against racism in the context of UK higher education[23,42-43]. CRT-E theorists have explored racial inequality in admissions, curriculum and pedagogy, teaching and learning, institutional culture, campus racial climate, and policy and finance[42-49]. However, CRT-E has failed to interrogate the role mental health plays in racial inequality. The Office for Students[2] stated that "black students with mental health conditions are being failed throughout the student cycle" (pg. 6), evidenced by their data which shows Black full-time students who report a mental health condition have some of the lowest attainment, continuation, and progression rates[2]. To understand and begin to address these concerns, further research is required into the role historically anti-Black racist systems embedded within UK universities plays on the mental health experiences of, risk factors for and challenges faced by Black university students affecting their mental health, experiences, outcomes, and progression.

This review is very timely given the recent global Black Lives Matter movement and protests following the murder of George Floyd[50]. Growing conversations around the role race and

For peer review only - http://bmjopen.bmj.com/site/about/guidelines.xhtml

institutional racism play on the mental health of Black people motivated students and staff to write multiple open letters[51-56] demanding UK universities do more to tackle racism, be anti-racist, and diversify and decolonise their curricula and institutions. Students and staff are calling out for UK universities to provide racially and culturally appropriate mental health support for their Black students. However, to achieve this, further research is needed to interrogate the eurocentric, ethnocentric, and egocentric ways in which institutional policy, research, and mental health practices disadvantage different Black students from varying identities and social statuses across the university student lifecycle.

The literature largely demonstrates an interest in Black university student mental. However this is limited exploration into whether and how mental health experiences differ across different Black student identities (including gender, culture, ethnicity, nationality), social statuses, and the impact of migration on student mental health. This is important given the finding that Black students believe universities treat them as a monolithic group[30-31,33,38]. There is a knowledge gap on suicidality among Black students. A recent national study[57] found the risk of suicide is lower amongst Black university students compared to White students (risk ratio (RR) 0.53 (95%CI 0.32–0.88)). This finding needs to be investigated further. One article included in this review[30] discussed that mental health is either not spoken about or spoken about differently in the Black community compared to within the wider UK university community. More studies are needed on mental health language and literacy.

The results of this review can assist academic staff, researchers, funders, and policymakers to identify potential areas of priority in student mental health services design, development, and reform with Black students. Based on the review findings, the authors recommend further investigation into the following potential strategies: (a) in-person or virtual Black student mental health peer support groups; (b) decolonization of mental health support services; (c) race, racism, and mental health training for academic staff and mental health practitioners; (d) decolonization of the classroom environment and teaching and learning materials; (e) scholarships and support funds for Black students[58]; (f) involvement of family in mental health support; (g) improve knowledge and accessibility of available services; (h) train and employ Black mental health

For peer review only - http://bmjopen.bmj.com/site/about/guidelines.xhtml

professionals; (i) involve religious organisations and chaplains in mental health support; (j) targeted mental health support for international-status students.

Strengths and limitations

To our knowledge, this is the first review and thematic synthesis to summarise the available literature on the experiences that affect the mental health and mental wellbeing of Black students studying at UK universities. By summarising these results, we make this information more readily available to students, academic staff, policymakers, and researchers. An exhaustive literature search was conducted using multiple databases, and manual searching. Dissertations, blogs, and unpublished articles were included alongside published articles. Our analysis allowed us to extract multiple recurrent themes pertaining to Black students' mental health. The results focus on both the personal and institutional factors that influence the mental health of Black university students.

The review has the following limitations. Despite the study selection criteria, no quantitative studies were included as they did not report the mental health experiences of Black university students. Secondly, although database and manual searching were extensive, it is possible that relevant articles were missed due to inconsistent terminology for mental health and mental wellbeing and the racial category "Black". Thirdly, the authors were unable to fully capture Black students' experiences as the citations selected from the included articles are a sample of the participants' subjective experiences and the researchers' interpretations. Fourthly, articles which included Black, Asian, and minoritised ethnic participants did not always clearly differentiate citations and themes derived specifically from Black student responses. The mixed racial category was not clearly defined in some articles so students who were mixed Black heritage may have been missed in the analysis. Responses had to be drawn from participants' accounts disclosing their race. Finally, our analysis was limited to the 'results' and 'findings' sections of the selected articles; however, all sections of the articles were read to provide a deep understanding of the topic. We acknowledge that themes developed in this synthesis may be similar to findings reported in the included articles since it was not our intention to develop new interpretations or theories, and we wanted to stay close to the experiences reported by the participants in the included articles.

For peer review only - http://bmjopen.bmj.com/site/about/guidelines.xhtml

Conclusion

This review and thematic synthesis offers an overview of the experiences that affect the mental health and mental wellbeing of Black students studying at universities in the UK as reported in the available literature. Our results suggest that there are both personal and institutional factors that affect the mental health and mental wellbeing of Black students. Further research into how the learning, social, living, cultural, and physical environment of universities affects the mental health of Black university students; and the differences in the lived experiences of Black university students across different identities and social statuses would give valuable insights. The impact of institutional issues of racism and sexism on the mental health of Black students studying at UK higher education needs to be further researched and explicitly addressed by policymakers and key decision-makers within universities.

Twitter: @NkasiStoll

Acknowledgements: The authors would like to acknowledge Dionne Laporte for proof-reading the draft of this manuscript. NS carried out this work as part of a London Interdisciplinary Social Science Doctoral Training Partnership funded doctoral project.

Contributors: NS (lead author) conceived the study which was further refined by SH (joint senior author) and HL (joint senior author). NS developed the detailed methodology, undertook database searches and title and abstract review. Each study retained for full-text review was reviewed by NS. YY reviewed 50% of the full text. Discrepancies between NS and YY regarding inclusion or exclusion were resolved by SH. NS and YY made the critical appraisal of included studies. The thematic synthesis was conducted by NS and revised by YY, SH, HL. All authors (NS, SH, HL, YY, NB) reviewed, commented, and edited the manuscript and approved the final version.

Funding: NS was supported by Economic and Social Research Council [grant number ES/P000703/1] via the London Interdisciplinary Social Science Doctoral Training Partnership.

For peer review only

SH is partly supported by the Economic and Social Research Council Centre for Society and Mental Health at King's College London [ES/S012567/1] and by the National Institute for Health Research (NIHR) Maudsley Biomedical Research Centre at South London and Maudsley NHS Foundation Trust and King's College London [BRC-1215-20018]. The views expressed are those of the authors and not necessarily those of the NHS, the NIHR or the Department of Health and Social Care, Wellcome Trust, ESRC or King's College London.

HL currently receives funding for successful grants as a PI or co- PI: Wellcome Trust Institutional Strategic Support Fund [N/A]; King's Health Partner Multiple Long-Term Conditions Challenge Fund [N/A]; National Axial Spondyloarthritis Society [N/A]; UKRCI Medical Research Council [MR/S001255/1]; Medical Research Council [MR/R023697/1]; National Institute for Health Research [RP-PG-0610-10066]; Guy's and St. Thomas' Charity London [EFT151101]; and Versus Arthritis [N/A].

NB is partly supported by the Economic and Social Research Council funding for SMaRteN [ES/S00324X/1].

The funders had no involvement in the study design; collection, analysis and interpretation of the data; writing of the paper; or decision to submit the paper for publication.

Competing interests: None declared.

Patient and public involvement: The lead author is a Black Caribbean PhD student, and the second author is a Black African master's student. Both were involved in the design, conduct, reporting, and dissemination of this research.

Patient consent for publication: Not required.

Data availability statement: No data are available.

For peer review only - http://bmjopen.bmj.com/site/about/guidelines.xhtml

Ethical Approval Statement: Not Applicable. This study is a review, with no participants, and does not require ethical approval.

Figure Legend

Figure 1.	PRISMA flow diagram
Table 1.	Characteristics of included studies (in date order)
Table 2.	Critical Appraisal Skills Programme (CASP) Checklist for included articles (in date order)
Table 3.	Concept matrix for included studies (in date order)

References

1. Hubble, S. and Bolton, P. (2020). Support for students with mental health issues in higher education in England. Accessed from: https://commonslibrary.parliament.uk/
2. Office for Students (2019). Mental health: Are all students being properly supported? Accessed from: https://www.officeforstudents.org.uk/media/b3e6669e-5337-4caa-9553-049b3e8e7803/insight-brief-mental-health-are-all-students-being-properly-supported.pdf
3. Universities UK (2017). Higher Education in Facts and Figures. Accessed from: http://www.universitiesuk.ac.uk/facts-and-stats/data-and-analysis/Documents/higher-education-in-facts-and-figures-2017.pdf
4. Universities UK (2018). 'Minding our future: Starting a conversation about the support of student mental health'. Accessed from: https://www.universitiesuk. ac.uk/policy-and-analysis/reports/Pages/minding-ourfuture-starting-a-conversation-support-student-mentalhealth.aspx
5. Temple, P., Callender, C., Grove, L. & Kersh, N. (2016) Managing the student experience in English higher education: differing responses to market pressures. London Review of Education, 14 (1). pp. 33-46. DOI: 10.18546/LRE.14.1.05
6. Stallman, H. M. (2008). Prevalence of psychological distress in university students. Australian Family Physician, 37(8), 673– 677.

For peer review only - http://bmjopen.bmj.com/site/about/guidelines.xhtml

7. Friedlander, L., Reid, G., Shupak, N., & Cribbie, R. (2007). Social Support, Self-Esteem, and Stress as Predictors of Adjustment to University Among First-Year Undergraduates. Journal of College Student Development, 48(3) 259-274. https://doi.org/10.1353
8. Andrews, B. & Wilding J.M. (2004). The relation of depression and anxiety to life-stress and achievement in students. British Journal of Psychology, 95, 509-521.
9. Cooke, R., Bewick, B.M., Barkham, M., Bradley, M. & Audin, K. (2006). Measuring, monitoring and managing the psychological well-being of first year university students. British Journal of Guidance and Counselling, 34, 505-518
10. Chung, W. W., & Hudziak, J. J. (2017). The Transitional Age Brain: "The Best of Times and the Worst of Times". Child and adolescent psychiatric clinics of North America, 26(2), 157–175. https://doi.org/10.1016/j.chc.2016.12.017).
11. Kessler, R. C., Amminger, G. P., Aguilar-Gaxiola, S., Alonso, J., Lee, S., & Ustün, T. B. (2007). Age of onset of mental disorders: a review of recent literature. *Current opinion in psychiatry*, *20*(4), 359–364. https://doi.org/10.1097/YCO.0b013e32816ebc8c
12. Boden, J.M. & Foulds, J.A. (2016). Major depression and alcohol use disorder in adolescence: Does comorbidity lead to poorer outcomes of depression? Journal of Affective Disorders, 206, 287-293. https://doi.org/10.1016/j.jad.2016.09.004.
13. McGorry, P. D., Purcell, R., Goldstone, S., & Amminger, G. P. (2011). Age of onset and timing of treatment for mental and substance use disorders: implications for preventive intervention strategies and models of care. Current opinion in psychiatry, 24(4), 301–306. https://doi.org/10.1097/YCO.0b013e3283477a09
14. Hawton, K., Saunders, K. E., & O'Connor, R. C. (2012). Self-harm and suicide in adolescents. Lancet (London, England), 379(9834), 2373–2382. https://doi.org/10.1016/S0140-6736(12)60322-5).
15. Eskin, M., Voracek, M., Tran, T., Sun, J., Janghorbani, M., Giovanni Carta, M., … & Harlak, H. (2016). Suicidal Behavior and Psychological Distress in University Students: A 12-Nation Study. Archives of Suicide Research. Archives of Suicide Research, 20(3), 369-388. doi: 10.1080/13811118.2015.1054055
16. Higher Education Statistics Agency (2019). Students by level of study and academic year. Accessed from: https://www.hesa.ac.uk/data-and-analysis/students/chart-1
17. Neves, J. (2019). UK Engagement Survey 2019. Advance HE. Accessed from: https://www.advance-he.ac.uk/knowledge-hub/uk-engagement-survey-2019

For peer review only

18. Universities UK (2019). Black, Asian and Minority Ethnic student attainment at UK universities: #closingthegap. Acessed from: https://www.universitiesuk.ac.uk/policy-and-analysis/reports/Documents/2019/bame-student-attainment-uk-universities-closing-the-gap.pdf

19. Livesey, A (2019) BAME underrepresentation in UK universities: a view from the humanities. Innovations in Practice, 13 (2) 11-17 http://dx.doi.org/10.24377/LJMU.iip.vol13iss2artic...

20. Schucan Bird, K., Pitman, L. How diverse is your reading list? Exploring issues of representation and decolonisation in the UK. High Educ 79, 903–920 (2020). https://doi.org/10.1007/s10734-019-00446-9

21. Leading Routes. (2019). The broken pipeline: Barriers to Black PhD students accessing research council funding. UCL/Leading Routes

22. Smith, S. 2017. "Exploring the Black and Minority Ethnic (BME) Student Attainment Gap: What Did It Tell Us? Actions to Address Home BME Undergraduate Students' Degree Attainment." Journal of Perspectives in Applied Academic Practice 5: 48–57.)

23. Rollock, N. and Gillborn, D. (2011) Critical Race Theory (CRT), British Educational Research Association online resource. Available online at https://www.bera.ac.uk/publication/critical-race-theory-crt. Last accessed July 2020.

24. Tong, A., Flemming, K., McInnes, E., Oliver, S., & Craig, J. (2012). Enhancing transparency in reporting the synthesis of qualitative research: ENTREQ. *BMC medical research methodology*, *12*, 181. https://doi.org/10.1186/1471-2288-12-181

25. Morgan, R. L., Whaley, P., Thayer, K. A., & Schünemann, H. J. (2018). Identifying the PECO: A framework for formulating good questions to explore the association of environmental and other exposures with health outcomes. Environment international, 121(Pt 1), 1027–1031. https://doi.org/10.1016/j.envint.2018.07.015

26. Critical Appraisal Skills Programme (2018). CASP Qualitative Checklist. [online]. Accessed: https://casp-uk.net/wp-content/uploads/2018/01/CASP-Systematic-Review-Checklist_2018.pdf

27. Braun, V., & Clarke, V. (2006). Using thematic analysis in psychology. *Qualitative Research in Psychology, 3*(2), 77–101. https://doi.org/10.1191/1478088706qp063oa

28. Thomas, J., & Harden, A. (2008). Methods for the thematic synthesis of qualitative research in systematic reviews. *BMC medical research methodology*, *8*, 45. https://doi.org/10.1186/1471-2288-8-45

29. Moher D, Liberati A, Tetzlaff J, Altman DG, The PRISMA Group (2009). *P*referred *R*eporting *I*tems for *S*ystematic Reviews and *M*eta-*A*nalyses: The PRISMA Statement. PLoS Med 6(7): e1000097. doi:10.1371/journal.pmed1000097

For peer review only - http://bmjopen.bmj.com/site/about/guidelines.xhtml

30. Hayford, A. (2019). A Thematic Analysis Exploring BAME Students Perceptions of Accessing Support Services at University

31. Jones, C. (2017). Black and minority ethnic postgraduates and mental health.Accessed from: https://www.blogs.hss.ed.ac.uk/pubs-and-publications/2017/12/01/black-minority-ethnic-postgraduates-mental-health/

32. Alloh, F. T., Tait, D., & Taylor, C. (2018). Away from Home: A Qualitative Exploration of Health Experiences of Nigerian Students in a U.K. University. Journal of International Students, 8(1), 1–20. Social Science Premium Collection. https://doi.org/10.5281/zenodo.1101024

33. Arday, J. (2018). Understanding Mental Health: What Are the Issues for Black and Ethnic Minority Students at University? Social Sciences, 7(10). Social Science Premium Collection. https://doi.org/10.3390/socsci7100196

34. Jackson-Cole, D. (2019).Navigating Toward Success: Black and Minority Ethnic Students In Postgraduate Science, Technology, Engineering And Mathematics Courses In England. 317. Accessed from: https://repository.uel.ac.uk/download/0a1bf27b3ce062c7bc8fca4abf37529f82da68723e654815b29ba71b0b0ee117/2999353/2019_PhD_Jackson-Cole.pdf

35. Myrie, C. V. and Gannon, K. (2013). "Should I really be here?" Exploring the relationship between Black men's conceptions of wellbeing, subject positions and help-seeking behaviour. Diversity and Equality in Health and Care. 10 (1), pp. 1-22

36. Bunce, L., King, N., Saran, S., & Talib, N. (2019). Experiences of black and minority ethnic (BME) students in higher education: Applying self-determination theory to understand the BME attainment gap. Studies in Higher Education, 1–14. https://doi.org/10.1080/03075079.2019.1643305

37. Cummings, S. (2018). Attainment and identity in the face of dual oppression: Exploring the educational experiences of British females of Caribbean heritage. Accessed from: http://etheses.Whiterose.ac.uk/21340/

38. Akel, S. (2019). Insider-Outsider: The Role of Race in Shaping the Experiences of Black and Minority Ethnic Students. 58. Accessed from: https://www.gold.ac.uk/media/docs/reports/Insider-Outsider-Report-191008.pdf

39. Bhatti, S. (2019). A Mixed Methods Study Investigating Black British Women's Perceptions and Attitudes Of Body Image And Body Appreciation. 214. Acessed from: https://openaccess.city.ac.uk/id/eprint/23922/

For peer review only

For peer review only - http://bmjopen.bmj.com/site/about/guidelines.xhtml

40. Crittle, K. (2018). Adjusting to UK life as an African American student. University Wire. Social Science Premium Collection. https://search.proquest.com/docview/2139161092?accountid=11862
41. Baxe, K. (2018). NUS Black Students Conference 2018. Retrieved 14 July 2020, from: https://www.centralsu.com/single-post/2018/05/15/NUS-Black-Students-Conference-2018
42. Phillips, C. (2011) *Institutional racism and ethnic inequalities: an expanded multilevel framework. Journal of social policy*, 40 (01). pp. 173-192.
43. Arday, J., & Mirza, H. (Eds.). (2018). *Dismantling race in higher education*. Basingstoke: Palgrave.
44. Warmington, P. (2020). Critical race theory in England: impact and opposition, Identities, 27:1, 20-37, Doi.: 10.1080/1070289X.2019.1587907
45. Bhopal, K. & Pitkin, C. (2020) 'Same old story, just a different policy': race and policy making in higher education in the UK, Race Ethnicity and Education, 23:4, 530-547, DOI: 10.1080/13613324.2020.1718082
46. Doharty, N., Madriaga, M., & Joseph-Salisbury, R. (2020): The university went to 'decolonise' and all they brought back was lousy diversity double-speak! Critical race counter-stories from faculty of colour in 'decolonial' times, Educational Philosophy and Theory, DOI: 10.1080/00131857.2020.176960
47. Ledesma, M. C., & Calderón, D. (2015). Critical Race Theory in Education: A Review of Past Literature and a Look to the Future. *Qualitative Inquiry*, *21*(3), 206–222. https://doi.org/10.1177/1077800414557825
48. Chakrabarty, N., Roberts, L. & Preston, J. (2012). Critical Race Theory in England, Race Ethnicity and Education, 15:1, 1-3, Doi: 10.1080/13613324.2012.638860
49. Mirza, H. S. (2018). Racism in higher education: What then, can be done? In J. Arday, & H. S. Mirza (Eds.), Dismantling race in higher education: Racism, Whiteness and decolonising the academy (pp. 1–7). Palgrave Macmillan
50. BBC News (2020). George Floyd death: The mental health impact. Accessed from: https://www.bbc.co.uk/news/av/uk-england-london-53009872
51. Times Higher Education (2020). We are at a crucial moment to address racial justice in higher education. Accessed from: https://www.timeshighereducation.com/letters/we-are-crucial-moment-address-racial-justice-higher-education
52. BBC News (2020). Aberdeen University students detail racism experience in letter. Accessed from: https://www.bbc.co.uk/news/uk-scotland-north-east-orkney-shetland-53106494

For peer review only - http://bmjopen.bmj.com/site/about/guidelines.xhtml

53. Oxford SU (2020). Open Letter to the Vice Chancellor on structural anti-Blackness at Oxford University. Accessed from: https://www.oxfordsu.org/news/article/6013/Open-Letter-to-the-Vice-Chancellor-on-structural-anti-Blackness-at-Oxford-University/

54. Jones, A. (2020). Open letter to Adam Tickell addressing racial inequality. Accessed from: https://thetab.com/uk/sussex/2020/06/04/open-letter-to-adam-tickell-addressing-racial-inequality-41924

55. Crawley, S. (2020). Open letters demand University does more to tackle racism. Accessed from: https://www.varsity.co.uk/news/19541

56. Taylor, Y. (2020). Over 2,200 sign open letter to St Andrews Principal demanding action over diversity policies. Accessed from: https://thetab.com/uk/stand/2020/06/10/over-2200-sign-open-letter-to-st-andrews-principal-demanding-action-over-diversity-policies-22866

57. Gunnell, D., Caul, S., Appleby, L., John, A., & Hawton, K. (2020). The incidence of suicide in University students in England and Wales 2000/2001–2016/2017: Record linkage study. Journal of Affective Disorders, 261, 113-120. https://doi.org/10.1016/j.jad.2019.09.079

58. University of Portsmouth (2020). Scholarship and Support Fund for Black Students. Accessed from: https://www.port.ac.uk/about-us/alumni/donate/support-black-students

For peer review only - http://bmjopen.bmj.com/site/about/guidelines.xhtml

Figure 1 PRISMA flow diagram

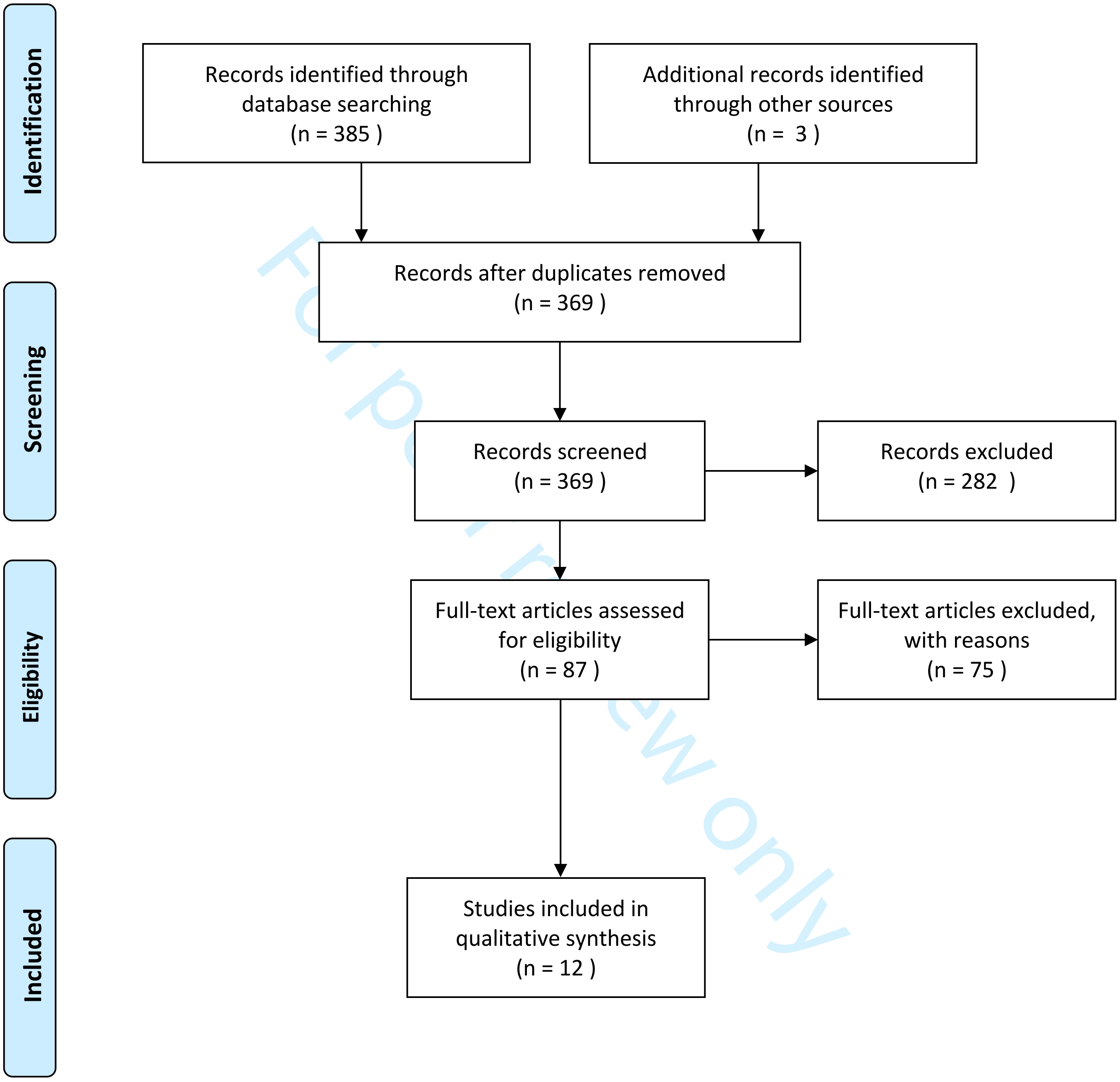

BMJ Open

Mental Health and Mental Wellbeing of Black Students at UK Universities: A Review and Thematic Synthesis

Journal:	*BMJ Open*
Manuscript ID	bmjopen-2021-050720.R2
Article Type:	Original research
Date Submitted by the Author:	15-Dec-2021

Complete List of Authors:	Stoll, Nkasi; King's College London Institute of Psychiatry Psychology and Neuroscience, Department of Psychological Medicine<br>Yalipende, Yannick; University College London, Division of Psychology and Language Sciences<br>Byrom, Nicola; King's College London Institute of Psychiatry Psychology and Neuroscience, Department of Psychology<br>HATCH, STEPHANI; King's College London Institute of Psychiatry Psychology and Neuroscience, Department of Psychological Medicine; King's College London, ESRC Centre for Society and Mental Health<br>Lempp, Heidi; King's College London Faculty of Life Sciences and Medicine, Department of Inflammation Biology

**Primary Subject Heading**:	Mental health
Secondary Subject Heading:	Mental health, Qualitative research, Sociology, Research methods, Health policy
Keywords:	MENTAL HEALTH, QUALITATIVE RESEARCH, PSYCHIATRY, PUBLIC HEALTH, SOCIAL MEDICINE, EPIDEMIOLOGY

SCHOLARONE™
Manuscripts

I, the Submitting Author has the right to grant and does grant on behalf of all authors of the Work (as defined in the below author licence), an exclusive licence and/or a non-exclusive licence for contributions from authors who are: i) UK Crown employees; ii) where BMJ has agreed a CC-BY licence shall apply, and/or iii) in accordance with the terms applicable for US Federal Government officers or employees acting as part of their official duties; on a worldwide, perpetual, irrevocable, royalty-free basis to BMJ Publishing Group Ltd ("BMJ") its licensees and where the relevant Journal is co-owned by BMJ to the co-owners of the Journal, to publish the Work in this journal and any other BMJ products and to exploit all rights, as set out in our licence.

The Submitting Author accepts and understands that any supply made under these terms is made by BMJ to the Submitting Author unless you are acting as an employee on behalf of your employer or a postgraduate student of an affiliated institution which is paying any applicable article publishing charge ("APC") for Open Access articles. Where the Submitting Author wishes to make the Work available on an Open Access basis (and intends to pay the relevant APC), the terms of reuse of such Open Access shall be governed by a Creative Commons licence – details of these licences and which Creative Commons licence will apply to this Work are set out in our licence referred to above.

Other than as permitted in any relevant BMJ Author's Self Archiving Policies, I confirm this Work has not been accepted for publication elsewhere, is not being considered for publication elsewhere and does not duplicate material already published. I confirm all authors consent to publication of this Work and authorise the granting of this licence.

For peer review only

Mental Health and Mental Wellbeing of Black Students at UK Universities: A Review and Thematic Synthesis

Ms Nkasi Stoll*; nkasi.1.stoll@kcl.ac.uk; King's College London, Institute of Psychiatry Psychology and Neuroscience, Department of Psychological Medicine, London, UK

E.319, Institute of Psychiatry Psychology and Neuroscience, Department of Psychological Medicine, Camberwell, London, UK, SE5 8AF

Mr Yannick Yalipende; yannick.yalipende.20@ucl.ac.uk; University College London, Division of Psychology and Language Sciences, London, UK

Dr Nicola Bryom; Nicola.byrom@kcl.ac.uk; King's College London, Institute of Psychiatry Psychology and Neuroscience, Department of Psychology, London, UK

Professor Stephani Hatch*; stephani.hatch@kcl.ac.uk; King's College London, Institute of Psychiatry Psychology and Neuroscience, Department of Psychological Medicine, London, UK; King's College London, ESRC Centre for Society and Mental Health, London, UK

Professor Heidi Lempp*; heidi.lempp@kcl.ac.uk; King's College London, Faculty of Life Sciences and Medicine, Department of Inflammation Biology, Centre for Rheumatic Diseases, London, UK

Five keyword(s): MENTAL HEALTH; QUALITATIVE RESEARCH; PSYCHIATRY; PUBLIC HEALTH; SOCIAL MEDICINE; EPIDEMIOLOGY

Word count: 3938

For peer review only - http://bmjopen.bmj.com/site/about/guidelines.xhtml

Abstract

Background: There is a knowledge gap about the experiences that affect the mental health of Black university students in the UK. Current research is focused on understanding the continuation, attainment and progression gap between Black students and non-Black students. It is essential to know more about the interactions between personal and institutional factors on the mental health of Black students to explain the inequalities in their experiences and outcomes across the university lifecycle. The current study set out to thematically synthesise articles that explore the experiences that affect the mental health and mental wellbeing of Black university students in the UK.

Methods: This study is a qualitative thematic synthesis of a literature review. We developed search strategies for four online databases (PubMed, Social Science Premium Collection via ProQuest, Open Access Theses and Dissertations, and Open Grey) covering January 2010 to July 2020. This search was combined with a manual search of reference lists and related citations. All articles in English addressing mental health and mental wellbeing experiences among Black university students studying at a UK university were included. Critical Appraisal Skills Programme (CASP) Checklist was used to assess bias. A thematic synthesis was conducted using Braun and Clarke (2006)'s six step guide to develop descriptive themes and analytical constructs.

Results: Twelve articles were included. Several themes were identified as affecting the mental health of Black university students in the UK: academic pressure, learning environment, Black gendered experience, isolation and alienation, culture shock, racism and support.

Discussion: This review provides an appraisal of the factors affecting the mental health and mental wellbeing of Black students at UK universities, which need to be addressed by Higher Education policymakers and key decision-makers. Further research is needed about the mental health experiences of Black university students in relation to Black identities, suicidality, mental health language, the physical environment, and racism and other institutional factors.

For peer review only - http://bmjopen.bmj.com/site/about/guidelines.xhtml

Strengths and Limitations of this study

- This is the first review and thematic synthesis to provide an overview of the personal and institutional experiences that affect the mental health and mental wellbeing of Black students studying at UK universities
- A thematic synthesis approach was used to describe and compare the main findings, to help inform policy and interventions
- Database and manual searching were extensive, screening 369 articles.
- It is possible not all relevant articles were found due to inconsistent terminology for the racial category "Black" and "Mixed" in existing studies.

For peer review only

Introduction

There is increasing concern about the mental health and mental wellbeing of university students in the United Kingdom (UK)[1-4]. The university student lifecycle has been conceptualised in terms of (i) the application experience which covers the interactions between potential students and the institution up to the point of arrival; (ii) the academic experience which encompasses students' interactions with their institution of study; (iii) the campus experience, which includes students' life not directly connected with study and may include activities away from the campus; and (iv) the graduate experience which covers the institution's role in assisting students' transition to employment or further study[5]. Throughout the lifecycle[5], British and international students are exposed to a range of experiences that make universities a high-risk period for maladaptive coping and the possible onset of poor mental health. These experiences include (but are not limited to): individuation, separation from family, new social connections, increased autonomy and responsibility, academic-related stress, financial concerns, sleep disruption, balancing conflicting demands of studying with personal and family life, and exposure to risky behaviours, including recreational drug use and alcohol binging[6-10]. Late adolescence through young adulthood is a peak age period for the first onset of mental health problems[11], which overlaps the period that many young adults enter and navigate the university student lifecycle[5]. Unsupported mental health problems are associated with progression to other co-morbid disorders, substance use disorders, self-harm, and suicide ideation, and attempts[12-15]. According to Higher Education Statistics Agency, there are 2.4 million university students in higher education in the UK[16]; which means that universities are in the best position to provide prevention and intervention to many young adults during a critical transitional period. In the UK, Black students are more likely than White students to self-report high levels of engagement and participation in their university studies; covering seven categories of engagement including course challenge, critical thinking, and research and inquiry [17]. Despite high engagement levels, Black students report lower satisfaction and are less likely to complete their course, achieve a first-class (70% or above) or upper second-class (60% or above) UK degree classification and progress to further education[18]. In addition to the aforementioned experiences and risk factors for poor mental health, there is tentative research around the institutional issues affecting Black university student

For peer review only - http://bmjopen.bmj.com/site/about/guidelines.xhtml

mental health including inequality in access, under-representation, the attainment gap, institutional racism, and racist curriculum[19-23]. To explain the inequalities in student experiences and outcomes across the university lifecycle, further understanding of the interactions between personal and institutional factors on the mental health of Black students' is needed. This review aims to synthesise the results of existing studies that explore mental health and mental wellbeing in Black students at UK universities. Therefore, our research question is as follows: what are the experiences that affect the mental health and mental wellbeing of Black university students in the UK, as reported in the literature?

Methods

Design

A review of the literature including quantitative, qualitative, and mixed studies was conducted on peer-reviewed and non-peer-reviewed articles, addressing the mental health and mental wellbeing of Black students at UK universities. Reporting of this review was guided by the Enhancing Transparency of Reporting the Synthesis of Qualitative Research framework [24], as the included articles were all qualitative.

Search methods

The following databases were searched for the period of January 2010 to July 2020: PubMed, Social Science Premium Collection via ProQuest, Open Access Theses and Dissertations, and Open Grey. The search strategy included several combinations of the following medical subject heading (MeSH) terms and keywords[25]: "mental health" or "psychological distress" or "mental wellbeing" and "University student" and "Black Caribbean" or "Black African" or "Black Mixed" or "Black ethnic*".

Data collection

For peer review only - http://bmjopen.bmj.com/site/about/guidelines.xhtml

All search results were saved to Zotero bibliographic management software and duplicates were removed. To be included in the review, articles had to (1) focus on the mental health or mental wellbeing of Black students at UK universities (2) apply empirical (e.g., quantitative, qualitative, or mixed-method research) or non-empirical research methods (e.g., theoretical, opinion-based, student transcripts, policy papers) (3) be written in English, (4) be conducted in the UK. The first author (NS) removed all articles that did not fit the inclusion criteria after full-text review. NS manually searched reference lists of eligible articles and contacted lead authors to search for unpublished literature. The authors decided to be lenient in the inclusion criteria during the screening and inclusion of articles because existing research on Black university student mental health is limited, and to enable the inclusion of articles that add value to the discussion of the topic. All included articles were critically appraised by NS and the author YY using the 'Critical Appraisal Skills Programme' (2018)[26] checklist for qualitative research.

Data analysis and synthesis

For each article, all text from 'Results/Findings' and 'Discussion' were extracted and imported into NVivo V.12 software (NVivo Qualitative data analysis Software; QSR International, V.12, 2018). Study characteristics were extracted into a Microsoft Excel grid by NS and revised by YY. Full-text screening, selection, data extraction, and critical appraisal of all articles was conducted prior to data analysis. A thematic analysis was conducted to describe and compare the main findings, following Braun and Clarke's (2006)[27] six-step guide. NS carefully read each article to familiarise themselves with the content, annotating initial ideas for codes and themes. NS then read and re-read the articles to develop an initial coding scheme, using a constant comparative approach[28]. Themes and sub-themes were identified to capture important patterns across and within the articles included. Six articles (50% of the sample) were randomly selected to be coded independently by YY, to provide validation. The codes were examined for similarities and differences and then organised into a hierarchy to create the final codes. Any differences were resolved by SH. The final codes were validated by NS, YY, SH, and HL, before being synthesized into the final thematic framework presented in this paper to minimise researcher subjectivity and improve the credibility of the work.

For peer review only - http://bmjopen.bmj.com/site/about/guidelines.xhtml

Patient and Public Involvement

The lead author is a Black Caribbean PhD student, and the second author is a Black African master's student. Both were involved in the design, conduct, reporting, and dissemination of this research.

Results

Included studies

Figure 1 shows the number of articles identified and rejected at each stage of the review process[29]. The search strategies identified 388 references of which 369 were kept after removing duplicates. After screening, 87 articles were read completely. Of these, 75 articles were excluded. A final sample of n=12 studies were included. Table 1 presents the characteristics of the included studies.

Table 1 Characteristics of included studies

Author(s)	Database	Type of article	No. of Black participants	Subject Characteristics	Location	Psychological phenomena explored	Methods
Hayford, A. (2019)	Hand searched	Dissertation	6	One was Caribbean, one was Somalian, one was Nigerian, two were mixed black African, and one was Ghanaian	East Anglia	Wellbeing	Interviews
Jones, C. (2017)[30]	PubMed	Blog	1	Black academic staff	UK	Wellbeing	Personal reflections and notes
Alloh, F. T., Tait, D., & Taylor, C. (2018)[31]	Proquest	Journal article	9	International Nigerian students studying at the master's-level in a U.K. university	southeast of England	Wellbeing	Interviews
Arday, J. (2018)[32]	Proquest	Journal article	14	32 BME university students	UK	Mental Health	Interviews focus Groups
Jackson-Cole, D. (2019)[33]	Open Grey	Doctoral thesis	10	Postgraduate students. two Black or Black British –	England	Wellbeing	Interviews

For peer review only - http://bmjopen.bmj.com/site/about/guidelines.xhtml

				Caribbean participants, seven Black or Black British –African, one Mixed –White and Black African			
Myrie, C. V. and Gannon, K. (2013)[34]	Hand searched	Journal article	3	2 Black African Students, 1 Black African and Caribbean Student	East and North London	Wellbeing	Interviews
Bunce, L., King, N., Saran, S., & Talib, N. (2019)[35]	PubMed	Journal article	14	14 full-time students from two degree programmes in health and social care related subjects - Black African (12) and White and Black Caribbean (2)	Unknown	Wellbeing	Focus Groups
Cummings, S. (2018)[36]	Open Grey	Doctoral thesis	5	Black women with Caribbean heritage, who were being educated at a university in the East Midlands	East Midlands	Wellbeing	Focus Groups
Akel, S. (2019)[37]	PubMed	Report	Not specified	195 BME students. 'Black' refers to people of African heritage and the diaspora.	London	Wellbeing	Interviews focus Groups
Bhatti, S. (2019)[38]	Open Grey	Doctoral thesis	5	Black students	London	Body image	Focus Groups
Crittle, K. (2018)[39]	Proquest	Blog	1	African American student	UK	Stress	Personal Reflections And Notes
Baxe, K. (2018)[40]	PubMed	Conference notes	1	Black students	UK	Mental Health	Personal Reflections And Notes

All included articles were assessed by NS and YY as reasonable quality and were therefore judged to have relevant contributions for the thematic synthesis (Table 2).

Table 2 Critical Appraisal Skills Programme (CASP) Checklist for included articles

For peer review only - http://bmjopen.bmj.com/site/about/guidelines.xhtml

	**Was there a clear statement of the aims of the research?**	**Is a qualitative methodology appropriate?**	**Was the research design appropriate to address the aims of the research?**	**Was the recruitment strategy appropriate to the aims of the research?**	**Was the data collected in a way that addressed the research issue?**	**Has the relationship between researcher and participants been adequately considered?**	**Have ethical issues been taken into consideration?**	**Was the data analysis sufficiently rigorous?**	**Is there a clear statement of findings?**	**How valuable is the research?**
Hayford, A. (2019)	Yes	Yes	Yes	Yes	Yes	Yes	Yes	Yes	Yes	Valuable
Jones, C. (2017)[30]	No	N/A	N/A	N/A	N/A	N/A	N/A	N/A	Yes	Valuable
Alloh, F. T., Tait, D., & Taylor, C. (2018)[31]	Yes	Yes	Yes	Yes	Yes	No	Yes	Yes	Yes	Valuable
Arday, J. (2018)[32]	Yes	Yes	Yes	Yes	Yes	No	Can't Tell	Yes	Yes	Valuable
Jackson-Cole, D. (2019)[33]	Yes	Yes	Yes	Yes	Yes	Yes	Yes	Yes	Yes	Valuable
Myrie, C. V. and Gannon, K. (2013)[34]	Yes	Yes	Yes	Yes	Yes	No	Yes	Yes	Yes	Valuable
Bunce, L., King, N., Saran, S., & Talib, N. (2019)[35]	Yes	Yes	Yes	Yes	Yes	No	Can't Tell	Yes	Yes	Valuable
Cummings, S. (2018)[36]	Yes	Yes	Yes	Yes	Yes	Yes	Yes	Yes	Yes	Valuable
Akel, S. (2019)[37]	Yes	Yes	Yes	Yes	Yes	No	Yes	Yes	Yes	Valuable
Bhatti, S. (2019)[38]	Yes	Yes	Yes	Yes	Yes	Yes	Yes	Yes	Yes	Valuable

For peer review only

Crittle, K. (2018)[39]	Yes	Yes	Yes	Yes	Yes	Yes	Yes	Yes	Yes	Valuable
Baxe, K. (2018)[40]	No	N/A	N/A	N/A	N/A	N/A	N/A	N/A	Yes	Valuable

Synthesised findings

The concept matrix for the findings can be seen in Table 3. We interpreted seven analytic themes derived from the participants' beliefs, perceptions, and experiences, and the article's authors' interpretations. The references for articles contributing to each of the themes are provided.

Table 3 Concept matrix for included studies

	**Support**	**Isolation and Alienation**	**Racism**	**Culture Shock**	**Black gendered experience**	**Learning environment**	**Academic pressure**
Hayford, A. (2019)	X	X		X			
Jones, C. (2017)[30]	X		X	X			X
Alloh, F. T., Tait, D., & Taylor, C. (2018)[31]	X		X	X			X
Arday, J. (2018)[32]	X	X	X		X		X
Jackson-Cole, D. (2019)[33]	X		X		X	X	
Myrie, C. V. and Gannon, K. (2013)[34]	X	X			X		
Bunce, L., King, N., Saran, S., & Talib, N. (2019)[35]		X		X		X	
Cummings, S. (2018)[36]	X	X	X		X	X	
Akel, S. (2019)[37]		X	X			X	
Bhatti, S. (2019)[38]	X				X		

For peer review only - http://bmjopen.bmj.com/site/about/guidelines.xhtml

Crittle, K. (2018)[39]	X	X					
Baxe, K. (2018)[40]		X					

Academic pressure

In six out of 12 articles, academic pressure was a determinant of mental distress for Black students[(Hayford, A. 2019)-34]. Students described their mental health as being adversely affected by the multiple and simultaneous academic tasks they were expected to complete during their degree[(Hayford, A. 2019)-30]. Cumulatively, Black students reported their observation that, unlike their non-Black peers, they faced pressures from their families and the wider Black community which meant they needed to work harder to succeed at university; which further exacerbated mental distress[(Hayford, A. 2019),30,32,33]. This pressure came from their families continuously reminding them of the sacrifices and investments made to support their education, specifically immigration and financial strain[(Hayford, A. 2019),30,34]. Black students with pre-existing mental and physical health conditions have been reported to be especially vulnerable to poor mental health caused by this academic pressure[30-31]. There was some evidence that Black students might not seek mental health support and instead prioritise their continuation and success in academia, at the expense of their mental health, as they feared a mental illness diagnosis might affect their success in academia[(Hayford, A. 2019),32].

Learning environment

In four of the 12 articles, researchers described Black students' perceptions of the university learning and teaching environment and how it impacted their mental health and mental wellbeing[33, 35-37]. Lectures that had discussions on diversity, inclusion, ethnicity, race, and identity were said to be cathartic and liberating for Black students, which contributed to positive wellbeing[35-37]. Black students reported they had to censor themselves in academic spaces to be seen by White students and staff as acceptable and agreeable instead of loud, disruptive, and confrontational[36]. This behaviour was believed to improve their learning experience and relationship with teaching staff, at the expense of their wellbeing. Participants spoke about their lack of relatedness to the White

majority academic staff, students, and teaching materials; and racism in the classroom made them feel excluded, frustrated, distressed, discouraged, and unmotivated to engage in their degree course[33,35-37]. In their university seminars, Black students recalled Black people were racially stereotyped as being bad people, criminals, and having lower intellectual ability by White students, and these stereotypes were sometimes reinforced by course materials[35-37]. Black female-identifying students recounted their academic knowledge was over-policed and over-scrutinised by teaching staff, and inaccessible learning and wellbeing support compared to men and White women on their course, which impacted their mental health and learning experience[33].

Black gendered experience

Black students' experiences of multiple marginalisation in the forms of racism and sexism was discussed in five articles[32-34,36,38]. Black male-identifying students reported being affected by the discourses of hyper-masculinity, which put pressure on them to not show their emotions, cope with their mental distress alone, and continue with their studies on their own[32,34]. This caused them to feel shame when seeking mental health support. For some female-identifying Black students, feelings about their health, ideal body shape, and size were influenced by the beliefs, culture, race, and ethnicity of the people in their lives[38]. The importance of seeing their body shape among Black students who looked like them was discussed[38]. Some students felt the White body ideal is not the same as the Black body ideal, therefore visiting their country of origin positively impacted their stress levels, self-esteem, and body image. Social media and the music industry were mentioned as vehicles in which the ideal Black female body is narrated, scrutinised, and picked apart[38].

Isolation and Alienation

Eight out of 12 included studies discussed isolation and alienation[(Hayford, A. 2019),32,34-37,39-40]. Black students who struggled with their mental health experienced stigma from their communities (including friends and family) before attending university, leaving them isolated from those communities[(Hayford, A. 2019)]. At university, participants reported being ignored or avoided by their non-Black peers in learning, social, and living environments which led to feelings of lack of

For peer review only - http://bmjopen.bmj.com/site/about/guidelines.xhtml

belonging, diminished their overall wellbeing, and evoked a range of negative emotions, including discomfort, distress, frustration, and anger[(Hayford, A. 2019),32,35-37,39-40]. Those with little or no mental health support from family felt isolated and alienated from their friends, family, peers, and academic staff, and used denial to cope with the lack of support, which exacerbated their mental health problems[32].

Black students reported that a lack of relatedness to White students affected their wellbeing, undermined their motivation for academic success, and had a negative influence on their sense of autonomy and competence[(Hayford, A. 2019),35-37]. Black students mentioned White students did not understand that they have to reconstruct their identity in the White image and practice silence to be successful at university and that this process negatively affected their mental health[(Hayford, A. 2019)].

Participants in one article discussed that academic staff do not always create safe and protective learning and living spaces for Black university students[37]. As a result, Black students reported they had to defend themselves by verbally challenging prejudicial beliefs shared in classrooms, which made White students uncomfortable, leaving Black students feeling bullied, alienated, and alone. Participants who had the support of other Black students in their learning environment at university reported being more comfortable, understood, and happy[35]. However, this may not apply to Black students who are international students. One article reported a student being in tears, feeling alone and isolated, and almost dropping out of university due to their African accent isolating them from Black British students and non-Black students[35].

Culture Shock

There was evidence suggesting Black international-status students had to adjust to new environments, cultures, and lifestyles in the UK which was characterised by changes in diet, weight, physical activity, sleep, alcohol, and smoking consumption, and led to psychological stress, depression, loneliness, and loss of confidence[(Hayford, A. 2019),31,35]. Black students who emigrated from a collectivist culture to the UK experienced culture shock during the transition from home to their new environment, that was characterised by worry, stress, and insomnia[31].

For peer review only

Contrastingly, there was evidence[31] to suggest Black home-status students had slightly poorer mental health and lower self-esteem than Black international-status students. Indeed, one article reported some Black international-status students believed their stress levels to be higher in their home country which gave them the perspective of living in the UK as being less stressful[31].

Racism

Five of the 12 included studies explored how institutional racism, discrimination, and hegemonic White privilege made universities toxic spaces for Black students, which affected their mental health and wellbeing[30, 32-33,36-37]. The constant stress of being confronted with racism (including racial microaggressions), discrimination, and having to survive hostile racist environments at university led to poor mental health and mental illness in Black students[30,32-33,36]. Racism had negative consequences on Black students' sense of belonging at university, motivation to socialise, their interactions with White students and staff, and their academic achievements and progression, which led to further mental distress. There was evidence to suggest Black students' recognition of racial difference had a greater impact on their mental wellbeing and potential or experienced mental health difficulties, than evidence that others treated them differently on account of their race[36]. Participants felt unable to express their feelings of racism and difference from White peers to others for fear they would not be understood, would be judged or rejected by peers and university staff, or due to uncertainty that these feelings were valid or legitimate[36]. These unarticulated feelings resulted in disengagement from university study and services.

Support

The aforementioned factors that possibly influence the mental health of Black students were discussed to affect and be affected by the type, access to, experience, and perceptions of mental health support in nine of the 12 included studies[(Hayford, A. 2019), 34, 36-39].

Black students reported they had to take care of their mental health by trying to remain positive and use distraction, utilise psycho-educational resources, change daily habits including exercise and nutrition, and use food to cope with stress at university and connect them to family[31-33, 36, 38].

For peer review only - http://bmjopen.bmj.com/site/about/guidelines.xhtml

Access to mental health support from family, friends, and peers was identified as a contributory factor to positive wellbeing and success at university[(Hayford, A. 2019)-30,32-33,36,39]. One article reported Black students who did not have familial or social capital found accessing university mental health support more difficult[32]. Four included articles[30,32,34,39] found mental health stigma left Black students feeling unsupported by their family and unable to get the professional support needed. Black-only spaces at university were reported as important to obtain support for mental health and wellbeing[(Hayford, A. 2019)-30,32,36,39]. These spaces gave students comfort, where they were not judged and misunderstood by non-Black students and staff; and could be with students from a shared background and experience. These Black-only spaces were especially important when there was a lack of Black students in classroom spaces, and could be online or in person. Black students also placed importance on mental health support from an understanding religious organisation or chaplain at their university[(Hayford, A. 2019)-30,32].

Included articles described Black students' beliefs that university services failed to provide appropriate support because they were institutionally racist and treated them like a monolithic group; and did not take responsibility for failing Black students [(Hayford, A. 2019)-30,32,37]. Participants' awareness that White mental health practitioners at university services were the majority race prevented them from accessing services, because of perceived racial stereotyping and lack of trust[32,37]. Some Black students avoided university mental health services to such an extent that they were not knowledgeable about what was available for them[(Hayford, A. 2019)]. Black students who needed mental health support did not seek such services until in crisis[(Hayford, A. 2019),32] and when they did, faced long waiting times[32]. Black students who did receive help from university mental health services reported experiencing racialised stereotypes that affected their relationship with White mental health practitioners[(Hayford, A. 2019)-30,32,37]. They had to educate practitioners on Black culture and the Black experience (which included how racism and micro-aggressions affected their mental health), which was a burden on their mental health and wellbeing[(Hayford, A. 2019)-30,32,37]. Because of this, students felt more comfortable with a Black, Asian or other minoritised ethnic mental health practitioner[(Hayford, A. 2019),32,37]. Black students described a sense of helplessness regarding their mental healthcare treatment[(Hayford, A. 2019),32,37]. One article discussed Black students felt unable to challenge mental health professionals about their healthcare treatment because they

For peer review only - http://bmjopen.bmj.com/site/about/guidelines.xhtml

may be labelled as difficult or unstable, and worsen their relationship with the mental healthcare provider[32].

Discussion

This review and thematic synthesis provides evidence on how the following experiences affect the mental health and mental wellbeing of Black university students in the UK: academic pressure, learning environment, Black gendered experience, isolation and alienation, culture shock, racism, and support.

The study findings highlight that for Black university students their mental health and mental wellbeing may be deeply rooted in institutional factors or dimensions, largely racism[30,32-33,36-38], which negatively affects most aspects of their higher education experiences, particularly in the learning, social, and living environments. Critical Race Theory in Education (CRT-E) is used to engage with and work against racism in the context of UK higher education[23,41-42]. CRT-E theorists have explored racial inequality in admissions, curriculum and pedagogy, teaching and learning, institutional culture, campus racial climate, and policy and finance[41-48]. However, CRT-E has failed to interrogate the role mental health plays in racial inequality. The Office for Students[2] stated that “Black students with mental health conditions are being failed throughout the student cycle” (pg. 6), evidenced by their data which shows Black full-time students who report a mental health condition have some of the lowest attainment, continuation, and progression rates[2]. To understand and begin to address these concerns, further research is required into the role historically anti-Black racist systems embedded within UK universities plays on the mental health experiences of, risk factors for and challenges faced by Black university students affecting their mental health, experiences, outcomes, and progression.

This review is very timely given the resurgence of the global Black Lives Matter movement and protests following the murder of George Floyd[49]. Growing conversations around the role race and institutional racism play on the mental health of Black people motivated students and staff to write multiple open letters[50-55] demanding UK universities do more to tackle racism, be anti-racist, and

For peer review only - http://bmjopen.bmj.com/site/about/guidelines.xhtml

diversify and decolonise their curricula and institutions. Students and staff are calling out for UK universities to provide racially and culturally appropriate mental health support for their Black students. However, to achieve this, further research is needed to interrogate the Eurocentric, ethnocentric, and egocentric ways in which institutional policy, research, and mental health practices disadvantage different Black students from varying identities and social statuses across the university student lifecycle.

The literature largely demonstrates an interest in Black university student mental health. However, this is limited exploration into whether and how mental health experiences differ across different Black student identities (including gender, culture, ethnicity, nationality), social statuses, and the impact of migration on student mental health. This is important given the finding that Black students believe universities treat them as a monolithic group[(Hayford, A. 2019)-30,32,37]. There is a knowledge gap on suicidality among Black students. A recent national study[56] found the risk of suicide is lower amongst Black university students compared to White students (risk ratio (RR) 0.53 (95%CI 0.32–0.88)). This finding needs to be investigated further. One article included in this review[(Hayford, A. 2019)] discussed that mental health is either not spoken about or spoken about differently in the Black community compared to within the wider UK university community. More studies are needed on mental health language and literacy.

The results of this review can assist academic staff, researchers, funders, and policymakers to identify potential areas of priority in student mental health services design, development, and reform with Black students. Based on the review findings, the authors recommend further investigation into the following potential strategies: (a) in-person or virtual Black student mental health peer support groups; (b) decolonization of mental health support services; (c) race, racism, and mental health training for academic staff and mental health practitioners; (d) decolonization of the classroom environment and teaching and learning materials; (e) scholarships and support funds for Black students[57]; (f) involvement of family in mental health support; (g) improve knowledge and accessibility of available services; (h) train and employ Black mental health professionals; (i) involve religious organisations and chaplains in mental health support; (j) targeted mental health support for international-status students.

For peer review only - http://bmjopen.bmj.com/site/about/guidelines.xhtml

Strengths and limitations

To our knowledge, this is the first review and thematic synthesis to summarise the available literature on the experiences that affect the mental health and mental wellbeing of Black students studying at UK universities. By summarising these results, we make this information more readily available to students, academic staff, policymakers, and researchers. An exhaustive literature search was conducted using multiple databases, and manual searching. Dissertations, blogs, and unpublished articles were included alongside published articles. Our analysis allowed us to extract multiple recurrent themes pertaining to Black students' mental health. The results focus on both the personal and institutional factors that influence the mental health of Black university students.

The review has the following limitations. Despite the study selection criteria, no quantitative studies were included as they did not report the mental health experiences of Black university students. Secondly, although database and manual searching were extensive, it is possible relevant articles were missed due to inconsistent terminology for mental health and mental wellbeing and the racial category "Black". Thirdly, the authors were unable to fully capture Black students' experiences as the citations selected from the included articles are a sample of the participants' subjective experiences and the researchers' interpretations. Fourthly, articles that included Black, Asian, and minoritised ethnic participants did not always clearly differentiate citations and themes derived specifically from Black student responses. The mixed racial category was not clearly defined in some articles so students who were of mixed Black heritage may have been missed in the analysis. Responses had to be drawn from participants' accounts disclosing their race. Finally, our analysis was limited to the 'results' and 'findings' sections of the selected articles; however, all sections of the articles were read to provide a deep understanding of the topic. We acknowledge that themes developed in this synthesis may be similar to findings reported in the included articles since it was not our intention to develop new interpretations or theories, and we wanted to stay close to the experiences reported by the participants in the included articles.

For peer review only - http://bmjopen.bmj.com/site/about/guidelines.xhtml

Conclusion

This review and thematic synthesis offer an overview of the experiences that affect the mental health and mental wellbeing of Black students studying at universities in the UK as reported in the available literature. Our results suggest that there are both personal and institutional factors that affect the mental health and mental wellbeing of Black students. Further research into how the learning, social, living, cultural, and physical environment of universities affects the mental health of Black university students; and the differences in the lived experiences of Black university students across different identities and social statuses would give valuable insights. The impact of institutional issues of racism and sexism on the mental health of Black students studying at UK higher education needs to be further researched and explicitly addressed by policymakers and key decision-makers within universities.

Twitter: @NkasiStoll

Acknowledgements: The authors would like to acknowledge Dionne Laporte for proof-reading the draft of this manuscript. NS carried out this work as part of a London Interdisciplinary Social Science Doctoral Training Partnership funded doctoral project.

Contributors: NS (lead author) conceived the study which was further refined by SH (joint senior author) and HL (joint senior author). NS developed the detailed methodology, undertook database searches and title and abstract review. Each study retained for full-text review was reviewed by NS. YY reviewed 50% of the full text. Discrepancies between NS and YY regarding inclusion or exclusion were resolved by SH. NS and YY made the critical appraisal of included studies. The thematic synthesis was conducted by NS and revised by YY, SH, HL. All authors (NS, SH, HL, YY, NB) reviewed, commented, and edited the manuscript and approved the final version.

Funding: NS was supported by Economic and Social Research Council [grant number ES/P000703/1] via the London Interdisciplinary Social Science Doctoral Training Partnership.

For peer review only - http://bmjopen.bmj.com/site/about/guidelines.xhtml

SH is partly supported by the Economic and Social Research Council Centre for Society and Mental Health at King's College London [ES/S012567/1] and by the National Institute for Health Research (NIHR) Maudsley Biomedical Research Centre at South London and Maudsley NHS Foundation Trust and King's College London [BRC-1215-20018]. The views expressed are those of the authors and not necessarily those of the NHS, the NIHR or the Department of Health and Social Care, Wellcome Trust, ESRC or King's College London.

HL currently receives funding for successful grants as a PI or co- PI: Wellcome Trust Institutional Strategic Support Fund [N/A]; King's Health Partner Multiple Long-Term Conditions Challenge Fund [N/A]; National Axial Spondyloarthritis Society [N/A]; UKRCI Medical Research Council [MR/S001255/1]; Medical Research Council [MR/R023697/1]; National Institute for Health Research [RP-PG-0610-10066]; Guy's and St. Thomas' Charity London [EFT151101]; and Versus Arthritis [N/A].

NB is partly supported by the Economic and Social Research Council funding for SMaRteN [ES/S00324X/1].

The funders had no involvement in the study design; collection, analysis and interpretation of the data; writing of the paper; or decision to submit the paper for publication.

Competing interests: None declared.

Patient and public involvement: The lead author is a Black Caribbean PhD student, and the second author is a Black African master's student. Both were involved in the design, conduct, reporting, and dissemination of this research.

Patient consent for publication: Not required.

Data availability statement: No data are available.

For peer review only - http://bmjopen.bmj.com/site/about/guidelines.xhtml

Ethical Approval Statement: Not Applicable. This study is a review, with no participants, and does not require ethical approval.

Figure Legend

Figure 1.	PRISMA flow diagram
Table 1.	Characteristics of included studies (in date order)
Table 2.	Critical Appraisal Skills Programme (CASP) Checklist for included articles (in date order)
Table 3.	Concept matrix for included studies (in date order)

References

1. Hubble, S. and Bolton, P. (2020). Support for students with mental health issues in higher education in England. Accessed from: https://commonslibrary.parliament.uk/
2. Office for Students (2019). Mental health: Are all students being properly supported? Accessed from: https://www.officeforstudents.org.uk/media/b3e6669e-5337-4caa-9553-049b3e8e7803/insight-brief-mental-health-are-all-students-being-properly-supported.pdf
3. Universities UK (2017). Higher Education in Facts and Figures. Accessed from: http://www.universitiesuk.ac.uk/facts-and-stats/data-and-analysis/Documents/higher-education-in-facts-and-figures-2017.pdf
4. Universities UK (2018). 'Minding our future: Starting a conversation about the support of student mental health'. Accessed from: https://www.universitiesuk. ac.uk/policy-and-analysis/reports/Pages/minding-ourfuture-starting-a-conversation-support-student-mentalhealth.aspx
5. Temple, P., Callender, C., Grove, L. & Kersh, N. (2016) Managing the student experience in English higher education: differing responses to market pressures. London Review of Education, 14 (1). pp. 33-46. DOI: 10.18546/LRE.14.1.05
6. Stallman, H. M. (2008). Prevalence of psychological distress in university students. Australian Family Physician, 37(8), 673– 677.

For peer review only - http://bmjopen.bmj.com/site/about/guidelines.xhtml

7. Friedlander, L., Reid, G., Shupak, N., & Cribbie, R. (2007). Social Support, Self-Esteem, and Stress as Predictors of Adjustment to University Among First-Year Undergraduates. Journal of College Student Development, 48(3) 259-274. https://doi.org/10.1353
8. Andrews, B. & Wilding J.M. (2004). The relation of depression and anxiety to life-stress and achievement in students. British Journal of Psychology, 95, 509-521.
9. Cooke, R., Bewick, B.M., Barkham, M., Bradley, M. & Audin, K. (2006). Measuring, monitoring and managing the psychological well-being of first year university students. British Journal of Guidance and Counselling, 34, 505-518
10. Chung, W. W., & Hudziak, J. J. (2017). The Transitional Age Brain: "The Best of Times and the Worst of Times". Child and adolescent psychiatric clinics of North America, 26(2), 157–175. https://doi.org/10.1016/j.chc.2016.12.017).
11. Kessler, R. C., Amminger, G. P., Aguilar-Gaxiola, S., Alonso, J., Lee, S., & Ustün, T. B. (2007). Age of onset of mental disorders: a review of recent literature. *Current opinion in psychiatry*, *20*(4), 359–364. https://doi.org/10.1097/YCO.0b013e32816ebc8c
12. Boden, J.M. & Foulds, J.A. (2016). Major depression and alcohol use disorder in adolescence: Does comorbidity lead to poorer outcomes of depression? Journal of Affective Disorders, 206, 287-293. https://doi.org/10.1016/j.jad.2016.09.004.
13. McGorry, P. D., Purcell, R., Goldstone, S., & Amminger, G. P. (2011). Age of onset and timing of treatment for mental and substance use disorders: implications for preventive intervention strategies and models of care. Current opinion in psychiatry, 24(4), 301–306. https://doi.org/10.1097/YCO.0b013e3283477a09
14. Hawton, K., Saunders, K. E., & O'Connor, R. C. (2012). Self-harm and suicide in adolescents. Lancet (London, England), 379(9834), 2373–2382. https://doi.org/10.1016/S0140-6736(12)60322-5).
15. Eskin, M., Voracek, M., Tran, T., Sun, J., Janghorbani, M., Giovanni Carta, M., … & Harlak, H. (2016). Suicidal Behavior and Psychological Distress in University Students: A 12-Nation Study. Archives of Suicide Research. Archives of Suicide Research, 20(3), 369-388. doi: 10.1080/13811118.2015.1054055
16. Higher Education Statistics Agency (2019). Students by level of study and academic year. Accessed from: https://www.hesa.ac.uk/data-and-analysis/students/chart-1
17. Neves, J. (2019). UK Engagement Survey 2019. Advance HE. Accessed from: https://www.advance-he.ac.uk/knowledge-hub/uk-engagement-survey-2019

For peer review only - http://bmjopen.bmj.com/site/about/guidelines.xhtml

18. Universities UK (2019). Black, Asian and Minority Ethnic student attainment at UK universities: #closingthegap. Acessed from: https://www.universitiesuk.ac.uk/policy-and-analysis/reports/Documents/2019/bame-student-attainment-uk-universities-closing-the-gap.pdf

19. Livesey, A (2019) BAME underrepresentation in UK universities: a view from the humanities. Innovations in Practice, 13 (2) 11-17 http://dx.doi.org/10.24377/LJMU.iip.vol13iss2artic...

20. Schucan Bird, K., Pitman, L. How diverse is your reading list? Exploring issues of representation and decolonisation in the UK. High Educ 79, 903–920 (2020). https://doi.org/10.1007/s10734-019-00446-9

21. Leading Routes. (2019). The broken pipeline: Barriers to Black PhD students accessing research council funding. UCL/Leading Routes

22. Smith, S. 2017. "Exploring the Black and Minority Ethnic (BME) Student Attainment Gap: What Did It Tell Us? Actions to Address Home BME Undergraduate Students' Degree Attainment." Journal of Perspectives in Applied Academic Practice 5: 48–57.)

23. Rollock, N. and Gillborn, D. (2011) Critical Race Theory (CRT), British Educational Research Association online resource. Available online at https://www.bera.ac.uk/publication/critical-race-theory-crt. Last accessed July 2020.

24. Tong, A., Flemming, K., McInnes, E., Oliver, S., & Craig, J. (2012). Enhancing transparency in reporting the synthesis of qualitative research: ENTREQ. *BMC medical research methodology*, *12*, 181. https://doi.org/10.1186/1471-2288-12-181

25. Morgan, R. L., Whaley, P., Thayer, K. A., & Schünemann, H. J. (2018). Identifying the PECO: A framework for formulating good questions to explore the association of environmental and other exposures with health outcomes. Environment international, 121(Pt 1), 1027–1031. https://doi.org/10.1016/j.envint.2018.07.015

26. Critical Appraisal Skills Programme (2018). CASP Qualitative Checklist. [online]. Accessed: https://casp-uk.net/wp-content/uploads/2018/01/CASP-Systematic-Review-Checklist_2018.pdf

27. Braun, V., & Clarke, V. (2006). Using thematic analysis in psychology. *Qualitative Research in Psychology, 3*(2), 77–101. https://doi.org/10.1191/1478088706qp063oa

28. Thomas, J., & Harden, A. (2008). Methods for the thematic synthesis of qualitative research in systematic reviews. *BMC medical research methodology*, *8*, 45. https://doi.org/10.1186/1471-2288-8-45

29. Moher D, Liberati A, Tetzlaff J, Altman DG, The PRISMA Group (2009). *P*referred *R*eporting *I*tems for *S*ystematic Reviews and *M*eta-*A*nalyses: The PRISMA Statement. PLoS Med 6(7): e1000097. doi:10.1371/journal.pmed1000097

For peer review only - http://bmjopen.bmj.com/site/about/guidelines.xhtml

30. Jones, C. (2017). Black and minority ethnic postgraduates and mental health. Accessed from: https://www.blogs.hss.ed.ac.uk/pubs-and-publications/2017/12/01/black-minority-ethnic-postgraduates-mental-health/
31. Alloh, F. T., Tait, D., & Taylor, C. (2018). Away from Home: A Qualitative Exploration of Health Experiences of Nigerian Students in a U.K. University. Journal of International Students, 8(1), 1–20. Social Science Premium Collection. https://doi.org/10.5281/zenodo.1101024
32. Arday, J. (2018). Understanding Mental Health: What Are the Issues for Black and Ethnic Minority Students at University? Social Sciences, 7(10). Social Science Premium Collection. https://doi.org/10.3390/socsci7100196
33. Jackson-Cole, D. (2019).Navigating Toward Success: Black and Minority Ethnic Students In Postgraduate Science, Technology, Engineering And Mathematics Courses In England. 317. Accessed from: https://repository.uel.ac.uk/download/0a1bf27b3ce062c7bc8fca4abf37529f82da68723e654815b29ba71b0b0ee117/2999353/2019_PhD_Jackson-Cole.pdf
34. Myrie, C. V. and Gannon, K. (2013). "Should I really be here?" Exploring the relationship between Black men's conceptions of wellbeing, subject positions and help-seeking behaviour. Diversity and Equality in Health and Care. 10 (1), pp. 1-22
35. Bunce, L., King, N., Saran, S., & Talib, N. (2019). Experiences of black and minority ethnic (BME) students in higher education: Applying self-determination theory to understand the BME attainment gap. Studies in Higher Education, 1–14. https://doi.org/10.1080/03075079.2019.1643305
36. Cummings, S. (2018). Attainment and identity in the face of dual oppression: Exploring the educational experiences of British females of Caribbean heritage. Accessed from: http://etheses.Whiterose.ac.uk/21340/
37. Akel, S. (2019). Insider-Outsider: The Role of Race in Shaping the Experiences of Black and Minority Ethnic Students. 58. Accessed from: https://www.gold.ac.uk/media/docs/reports/Insider-Outsider-Report-191008.pdf
38. Bhatti, S. (2019). A Mixed Methods Study Investigating Black British Women's Perceptions and Attitudes Of Body Image And Body Appreciation. 214. Accessed from: https://openaccess.city.ac.uk/id/eprint/23922/
39. Crittle, K. (2018). Adjusting to UK life as an African American student. University Wire. Social Science Premium Collection. https://search.proquest.com/docview/2139161092?accountid=11862

For peer review only - http://bmjopen.bmj.com/site/about/guidelines.xhtml

40. Baxe, K. (2018). NUS Black Students Conference 2018. Retrieved 14 July 2020, from: https://www.centralsu.com/single-post/2018/05/15/NUS-Black-Students-Conference-2018

41. Phillips, C. (2011) *Institutional racism and ethnic inequalities: an expanded multilevel framework. Journal of social policy*, 40 (01). pp. 173-192.

42. Arday, J., & Mirza, H. (Eds.). (2018). *Dismantling race in higher education*. Basingstoke: Palgrave.

43. Warmington, P. (2020). Critical race theory in England: impact and opposition, Identities, 27:1, 20-37, Doi.: 10.1080/1070289X.2019.1587907

44. Bhopal, K. & Pitkin, C. (2020) 'Same old story, just a different policy': race and policy making in higher education in the UK, Race Ethnicity and Education, 23:4, 530-547, DOI: 10.1080/13613324.2020.1718082

45. Doharty, N., Madriaga, M., & Joseph-Salisbury, R. (2020): The university went to 'decolonise' and all they brought back was lousy diversity double-speak! Critical race counter-stories from faculty of colour in 'decolonial' times, Educational Philosophy and Theory, DOI: 10.1080/00131857.2020.176960

46. Ledesma, M. C., & Calderón, D. (2015). Critical Race Theory in Education: A Review of Past Literature and a Look to the Future. *Qualitative Inquiry*, *21*(3), 206–222. https://doi.org/10.1177/1077800414557825

47. Chakrabarty, N., Roberts, L. & Preston, J. (2012). Critical Race Theory in England, Race Ethnicity and Education, 15:1, 1-3, Doi: 10.1080/13613324.2012.638860

48. Mirza, H. S. (2018). Racism in higher education: What then, can be done? In J. Arday, & H. S. Mirza (Eds.), Dismantling race in higher education: Racism, Whiteness and decolonising the academy (pp. 1–7). Palgrave Macmillan

49. BBC News (2020). George Floyd death: The mental health impact. Accessed from: https://www.bbc.co.uk/news/av/uk-england-london-53009872

50. Times Higher Education (2020). We are at a crucial moment to address racial justice in higher education. Accessed from: https://www.timeshighereducation.com/letters/we-are-crucial-moment-address-racial-justice-higher-education

51. BBC News (2020). Aberdeen University students detail racism experience in letter. Accessed from: https://www.bbc.co.uk/news/uk-scotland-north-east-orkney-shetland-53106494

52. Oxford SU (2020). Open Letter to the Vice Chancellor on structural anti-Blackness at Oxford University. Accessed from: https://www.oxfordsu.org/news/article/6013/Open-Letter-to-the-Vice-Chancellor-on-structural-anti-Blackness-at-Oxford-University/

For peer review only

53. Jones, A. (2020). Open letter to Adam Tickell addressing racial inequality. Accessed from: https://thetab.com/uk/sussex/2020/06/04/open-letter-to-adam-tickell-addressing-racial-inequality-41924

54. Crawley, S. (2020). Open letters demand University does more to tackle racism. Accessed from: https://www.varsity.co.uk/news/19541

55. Taylor, Y. (2020). Over 2,200 sign open letter to St Andrews Principal demanding action over diversity policies. Accessed from: https://thetab.com/uk/stand/2020/06/10/over-2200-sign-open-letter-to-st-andrews-principal-demanding-action-over-diversity-policies-22866

56. Gunnell, D., Caul, S., Appleby, L., John, A., & Hawton, K. (2020). The incidence of suicide in University students in England and Wales 2000/2001–2016/2017: Record linkage study. Journal of Affective Disorders, 261, 113-120. https://doi.org/10.1016/j.jad.2019.09.079

57. University of Portsmouth (2020). Scholarship and Support Fund for Black Students. Accessed from: https://www.port.ac.uk/about-us/alumni/donate/support-black-students

For peer review only

Figure 1 PRISMA flow diagram

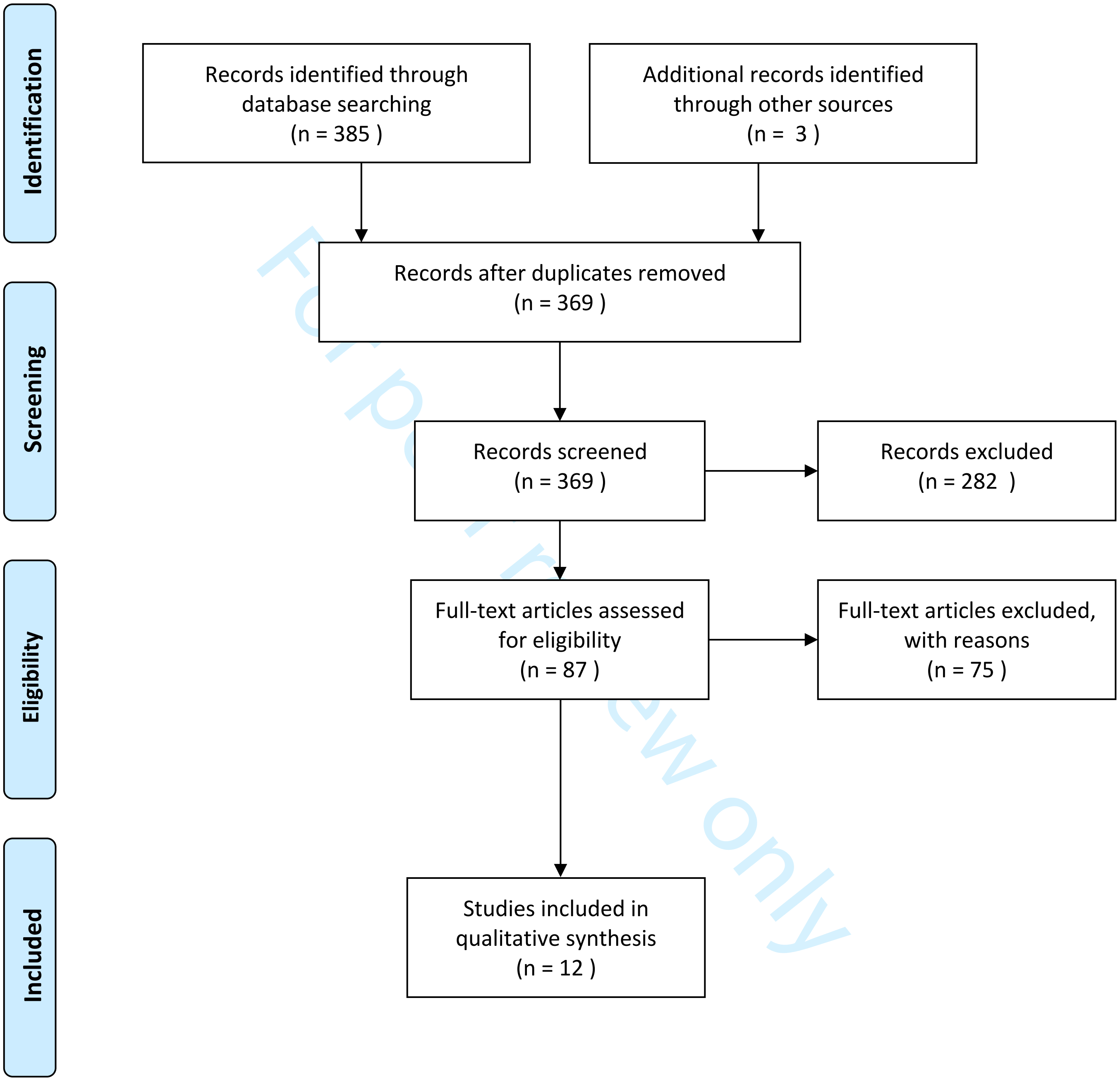

PRISMA 2009 Checklist

Section/topic	#	Checklist item	Reported on page #
**TITLE**			
Title	1	Identify the report as a systematic review, meta-analysis, or both.	1
**ABSTRACT**			
Structured summary	2	Provide a structured summary including, as applicable: background; objectives; data sources; study eligibility criteria, participants, and interventions; study appraisal and synthesis methods; results; limitations; conclusions and implications of key findings; systematic review registration number.	2-3
**INTRODUCTION**			
Rationale	3	Describe the rationale for the review in the context of what is already known.	4-5
Objectives	4	Provide an explicit statement of questions being addressed with reference to participants, interventions, comparisons, outcomes, and study design (PICOS).	5
**METHODS**			
Protocol and registration	5	Indicate if a review protocol exists, if and where it can be accessed (e.g., Web address), and, if available, provide registration information including registration number.	N/A
n	6	Specify study characteristics (e.g., PICOS, length of follow-up) and report characteristics (e.g., years considered, language, publication status) used as criteria for eligibility, giving rationale.	5-6
Information sources	7	Describe all information sources (e.g., databases with dates of coverage, contact with study authors to identify additional studies) in the search and date last searched.	5
Search	8	Present full electronic search strategy for at least one database, including any limits used, such that it could be repeated.	5-6
Study selection	9	State the process for selecting studies (i.e., screening, eligibility, included in systematic review, and, if applicable, included in the meta-analysis).	6
Data collection process	10	Describe method of data extraction from reports (e.g., piloted forms, independently, in duplicate) and any processes for obtaining and confirming data from investigators.	6
Data items	11	List and define all variables for which data were sought (e.g., PICOS, funding sources) and any assumptions and simplifications made.	5-6 (Constructs not variables)
Risk of bias in individual studies	12	Describe methods used for assessing risk of bias of individual studies (including specification of whether this was done at the study or outcome level), and how this information is to be used in any data synthesis.	6
Summary measures	13	State the principal summary measures (e.g., risk ratio, difference in means).	N/A

For peer review only

PRISMA 2009 Checklist

Synthesis of results	14	Describe the methods of handling data and combining results of studies, if done, including measures of consistency (e.g., I^2) for each meta-analysis.	N/A – qual thematic synthesis approach

Section/topic	#	Checklist item	Reported on page #
Risk of bias across studies	15	Specify any assessment of risk of bias that may affect the cumulative evidence (e.g., publication bias, selective reporting within studies).	N/A
Additional analyses	16	Describe methods of additional analyses (e.g., sensitivity or subgroup analyses, meta-regression), if done, indicating which were pre-specified.	N/A
**RESULTS**			
Study selection	17	Give numbers of studies screened, assessed for eligibility, and included in the review, with reasons for exclusions at each stage, ideally with a flow diagram.	7<br>Supplementary file #1
Study characteristics	18	For each study, present characteristics for which data were extracted (e.g., study size, PICOS, follow-up period) and provide the citations.	7-8
Risk of bias within studies	19	Present data on risk of bias of each study and, if available, any outcome level assessment (see item 12).	9-10
Results of individual studies	20	For all outcomes considered (benefits or harms), present, for each study: (a) simple summary data for each intervention group (b) effect estimates and confidence intervals, ideally with a forest plot.	10-11 (concept matrix)
Synthesis of results	21	Present results of each meta-analysis done, including confidence intervals and measures of consistency.	11-15
Risk of bias across studies	22	Present results of any assessment of risk of bias across studies (see Item 15).	N/A
Additional analysis	23	Give results of additional analyses, if done (e.g., sensitivity or subgroup analyses, meta-regression [see Item 16]).	N/A
**DISCUSSION**			
Summary of evidence	24	Summarize the main findings including the strength of evidence for each main outcome; consider their relevance to key groups (e.g., healthcare providers, users, and policy makers).	16
Limitations	25	Discuss limitations at study and outcome level (e.g., risk of bias), and at review-level (e.g., incomplete retrieval of identified research, reporting bias).	17-18
Conclusions	26	Provide a general interpretation of the results in the context of other evidence, and implications for future research.	18-19
**FUNDING**			

For peer review only - http://bmjopen.bmj.com/site/about/guidelines.xhtml

PRISMA 2009 Checklist

Funding	27	Describe sources of funding for the systematic review and other support (e.g., supply of data); role of funders for the systematic review.	19-20

From: Moher D, Liberati A, Tetzlaff J, Altman DG, The PRISMA Group (2009). Preferred Reporting Items for Systematic Reviews and Meta-Analyses: The PRISMA Statement. PLoS Med 6(7): e1000097. doi:10.1371/journal.pmed1000097

For more information, visit: **www.prisma-statement.org**.

For peer review only

For peer review only - http://bmjopen.bmj.com/site/about/guidelines.xhtml
